# Surface Reconstruction as a Design Principle for Ni‐rich Cathodes

**DOI:** 10.1002/smsc.202500503

**Published:** 2025-12-10

**Authors:** Sumaiyatul Ahsan, Abiram Krishnan, Mengkun Tian, Samir Sarma, Faisal M. Alamgir

**Affiliations:** ^1^ School of Materials Science and Engineering Georgia Institute of Technology Atlanta GA 30332 USA; ^2^ Institute for Matter & Systems Georgia Institute of Technology Atlanta GA 30332 USA

**Keywords:** density functional theory, electrochemistry, Ni‐rich cathodes, surface reconstruction, X‐ray diffraction

## Abstract

Surface reconstruction by formation of inert phases in Ni‐rich cathodes is widely viewed as a degradation mechanism for batteries. Herein, this seemingly undesirable phase is leveraged to stabilize Ni‐rich cathodes. Density functional theory reveals a reduction in Ni 3d–O 2p hybridization in NiO compared to LiNiO_2_ (LNO), suggesting its potential as a protective layer. Guided by theory, variable temperature X‐ray diffraction is used to identify optimal conditions for introducing oxygen vacancies on the surface of LiNi_0.8_Mn_0.1_Co_0.1_O_2_ (NMC811) particles, which triggers a phase transformation from layered to rock‐salt NiO on the surface, creating a core–shell structure as evidenced by X‐ray photoelectron spectroscopy and scanning transmission electron microscopy (STEM). Electrochemical methods such as constant‐current long‐term cycling, cyclic voltammetry, and electrochemical impedance spectroscopy reveal improved capacity, higher Li^+^ diffusivity, and lower resistance during cycling. X‐ray absorption spectroscopy confirms that the bulk‐averaged oxidation state remains unchanged after modification, and STEM imaging confirm reduced structural heterogeneity. By reframing surface NiO as a controllable design principle, a materials‐intrinsic, scalable route to extend the durability of Ni‐rich cathodes is offered.

## Graphical abstract

A comprehensive understanding of how controlled surface reconstruction via oxygen vacancy‐induced NiO formation enhances the electrochemical performance of Ni‐rich cathodes is presented. This work reframes inert phase formation as a functional design principle, enabling scalable surface protection and improving the cycling performance of NMC811.

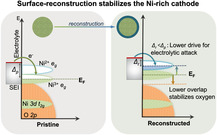

## 1 Introduction

Surface reconstruction in Ni‐rich layered cathodes is traditionally regarded as a hallmark of degradation.^[^ ^1^, ^2^, ^3^ ^]^ This reconstruction is characterized by the layered structure (R$\bar{3}$m) transforming into inert metal oxides such as spinel Ni_3_O_4_ (Fd$\bar{3}$m) or wide‐bandgap rock‐salt NiO (Fm$\bar{3}$m) phases.^[^ ^4^ ^]^ Yet, paradoxically, surface‐engineering strategies such as coating and doping routinely introduce inert phases, to enhance stability.^[^ ^5^, ^6^, ^7^ ^]^ Furthermore, wide‐bandgap metal oxides, such as Al_2_O_3_ are used to passivate the surface^[^ ^1^ ^]^ as they are less reactive with organic electrolytes. This raises a fundamental question: can the intrinsic wide‐bandgap phase, NiO also stabilize the surface?

Several challenges complicate this prospect. During cycling, surface phase transitions are accompanied by oxygen and lithium loss, fueling further phase transformation,^[^ ^8^ ^]^ which emphasizes the importance of forming the NiO phase prior to cycling. Furthermore, if inert phases nucleate within the bulk^[^ ^9^ ^]^ and not on the surface, they can serve as crack initiation sites. Hence, the phase transformation to NiO must be localized on the surface.

**Figure 1:**
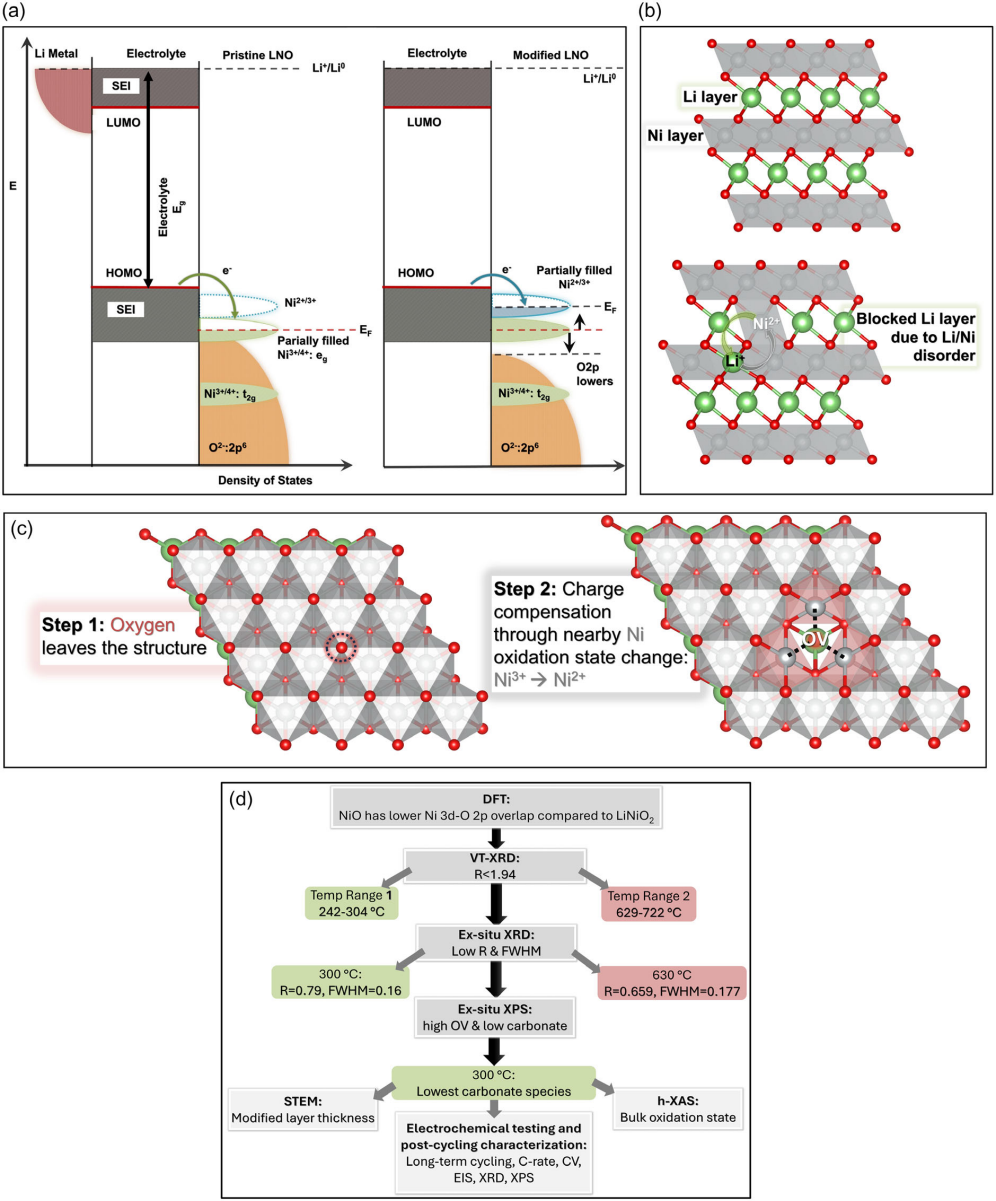
Electronic structure and defect‐driven stabilization of Ni‐rich cathodes. a) Schematic DOS diagrams for pristine and modified LiNiO_2_ (LNO), aligned to the Li^+^/Li^0^ reference. In pristine LNO, low‐lying Ni^3+/4+^ e_g_ states drive electrolyte oxidation and surface degradation. OVs shift Ni^2+/3+^ e_g_ levels closer to the HOMO, lower O 2p states, and reduce Ni—O covalency, suppressing parasitic reactions. b) Ni^2+^/Li^+^ interlayer mixing blocks Li^+^ diffusion channels during cycling. c) Surface OVs prevent cation disorder by stabilizing Ni^2+^. d) Schematic diagram illustrating the overall technical workflow.

The surface of LiNi_0.9_Co_0.1_O_2_ (NCA), engineered to be rich in dislocations alongside inert rock‐salt phases, has been shown to enhance electrochemical properties.^[^ ^10^ ^]^ However, the benefits of such modification have been solely attributed to the dislocation‐dense surface in annihilating crack propagation and not NiO formation. Despite these mechanical considerations, we argue that surface degradation in Ni‐rich cathodes is fundamentally an electrochemical phenomenon.^[^ ^11^ ^]^ As illustrated in **Figure** **1**a, in LiNiO_2_ (LNO), the Ni^3+/4+^ electronic states lie significantly below (≈0.4 eV) the highest occupied molecular orbital (HOMO) of typical electrolytes, creating a thermodynamic driving force for electron transfer from the electrolyte to the cathode resulting in electrolyte decomposition.^[^ ^12^ ^]^ The strong Ni—O hybridization accelerates oxygen evolution.^[^ ^13^ ^]^

By contrast, modified LNO with a higher surface %Ni^2+^ exhibits a partially filled Ni^2+/3+^ that aligns closely with the electrolyte HOMO and the O 2p band center shifts to lower energy due to lower Ni 3d–O 2p overlap, reducing the driving forces for surface degradation and oxygen degassing.

However, stabilizing Ni^2+^ presents an additional challenge. The ionic radius of Ni^2+^(0.69 Å) is similar to that of Li^+^(0.76 Å). Hence, Ni^2+^ migrates into the lithium layer and block Li‐ion transport (Figure 1b). Thus, Ni^2+^ must be structurally anchored to prevent migration.

We hypothesize that oxygen vacancies (OVs) offer a pathway to achieve this stabilization. As illustrated in Figure 1c, loss of oxygen renders (Step 1) neighboring Ni atoms undercoordinated, reducing the oxidation state from Ni^3+^ to Ni^2+^ (Step 2). Crucially, this locally coordinated Ni^2+^ becomes less mobile and effectively pinned within the structure. Finally, the metastable layered structure with OVs should result in a phase transformation to stable NiO.

To experimentally validate this hypothesis, we employed the simplest, scalable, single‐step thermal treatment to introduce ≈6.9% OVs in NMC811 prior to electrochemical cycling. This process forms a homogeneous rock‐salt phase that stabilizes the surface using the cathodes’ intrinsic chemistry without the need for complex synthesis involving foreign oxides,^[^ ^14^ ^]^ fluorides,^[^ ^15^ ^]^ heavy metals,^[^ ^16^ ^]^ etc. The thermal treatment also homogenizes the bulk phase into a near‐perfect layered structure, which is generally absent in pristine Ni‐rich cathodes. As a result, the modified NMC811 exhibits higher discharge capacity over 100 cycles at 1C, along with significant reduction in resistance during initial and after long‐term cycling. By embracing controlled surface reconstruction through intentional OV engineering, we transform a conventional degradation pathway into a design principle.

Rather than relying on conventional trial‐and‐error electrochemical testing^[^ ^10^ ^]^ across multiple annealing temperatures, this study adopts a hypothesis‐driven approach to identify the optimal OV formation window. A schematic overview of the study (Figure 1d) summarizes this integrated workflow. Using variable temperature X‐ray diffraction (VT‐XRD), we tracked structural evolution with temperature to pinpoint the onset of lattice distortion and oxygen release. Complementary ex situ XRD and X‐ray photoelectron spectroscopy (XPS) analyses were then used to refine this range, distinguishing between beneficial vacancy formation (OV300_NMC811) and excessive surface degradation (OV630_NMC811). This integrative approach enables a rational definition of the processing window that maximizes defect‐driven performance while maintaining structural integrity. Finally, STEM, hard X‐ray absorption spectroscopy (h‐XAS), electrochemical testing, and postcycling characterization were employed to elucidate the mechanisms underlying the improved cell performance. Together, this framework demonstrates how predictive modeling and targeted materials characterization can streamline the rational design of stable Ni‐rich cathodes.

## 2 Results and Discussion

### 2.1 Theoretical Basis for Modification

The extent of NiO formation varies across different Ni‐rich chemistries: LNO, NMC622, NMC811, and NCA. However, high Ni content generally leads to NiO formation.^[^ ^7^ ^]^ Hence, to computationally evaluate the potential benefits of NiO phase, we compare it to the simplest cathode chemistry, LNO to identify changes in electronic properties that may support intentional surface reconstruction.

**Figure 2:**
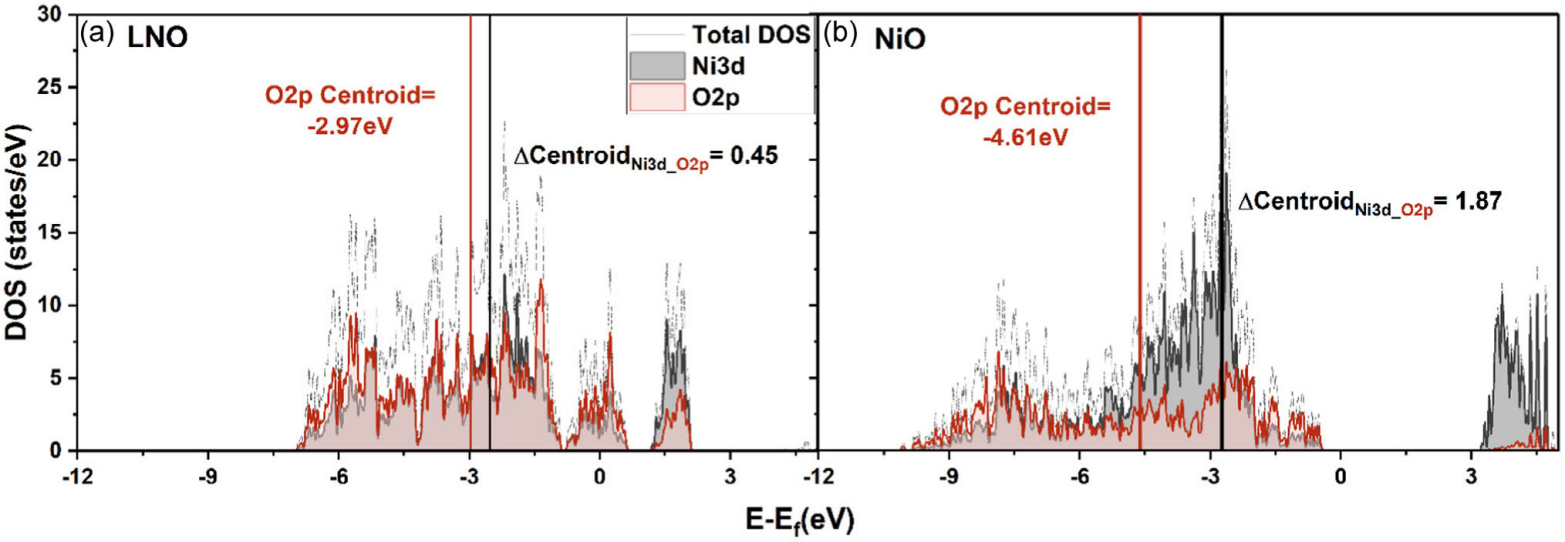
Total and PDOS of LiNiO_2_ (LNO) and NiO. a) LNO shows strong overlap between the Ni 3d and O 2p bands. b) NiO shows a higher Ni 3d–O 2p centroid separation of 1.87 eV, indicating significantly lower covalency.

Density functional theory (DFT) was used to simulate the total and projected density of states (DOS and PDOS) for LNO and NiO (**Figure** **2**a,b). In LNO (Figure 2a), the centroids of the Ni 3d and O 2p PDOS strongly overlap, with a centroid difference of only 0.45 eV, indicating a high degree of hybridization. This overlap underpins the covalency between Ni and O; hence, during delithiation, both Ni and O participate in charge compensation.^[^ ^17^ ^]^ Especially, at deeper delithiation,^[^ ^11^ ^]^ oxygen begins to play a more dominant role in redox activity via the following steps:

(1) $$\text{Step }1\;\left(\text{Anionic oxidation}\right):{\text{ O}}^{2-}\left(\text{LNO}\right)\to O^{-}+e^{-}\;\left(\text{extraction through external circuit}\right)$$

(2)

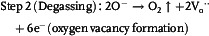




This oxygen loss leads to irreversible structural degradation and is not an artifact of processing conditions, but an intrinsic consequence of hybridization.

By contrast, NiO (Figure 2b) shows a significant reduction in Ni 3d–O 2p overlap, indicated by the centroid difference of 1.87 eV (Table S1, Supporting Information). This results in a weakening of Ni—O covalency and stabilization of Ni^2+^.

Furthermore, the O 2p centroid shifts from −2.97 eV (in LNO) to −4.61 eV (in NiO) below the Fermi energy, which minimizes the energetic driving force for O oxidation in NiO and intrinsically mitigates O_2_ evolution. Finally, Figure 2a,b illustrates that NiO is a wide‐bandgap antiferromagnetic insulator, whereas LNO is a ferromagnetic material with no bandgap (Figure S1, Supporting Information). The computationally measured bandgap for NiO was 3.41 eV, which matched with experimental literature.^[^ ^18^ ^]^ Notably, high bandgap materials are often used to passivate the surface of cathodes.^[^ ^1^, ^19^, ^20^ ^]^ This is done because metals and semiconductors (LNO) bind hydrogen more readily than materials with a larger bandgap (NiO), resulting in higher surface degradation when in contact with electrolyte.^[^ ^20^ ^]^ Which means LNO and other Ni‐rich cathodes,^[^ ^21^ ^]^ undergoes surface reduction more readily compared to NiO.

Hence, the benefit of the surface modification is twofold: lowering the O 2p energy relative to the Fermi energy and inducing a bandgap through the formation of NiO.

### 2.2 Surface Phase Transition Temperature

While past studies have explored heating‐based surface treatments for cathodes, researchers typically based the temperature selection through trial and error,^[^ ^10^ ^]^ or the temperature was selected to be high enough (≥600 °C) for uniform properties^[^ ^14^, ^22^ ^]^ or to remove surface impurities.^[^ ^19^ ^]^ Here, we systematically identify a lower temperature region for forming NiO on the surface of NMC811.

**Figure 3:**
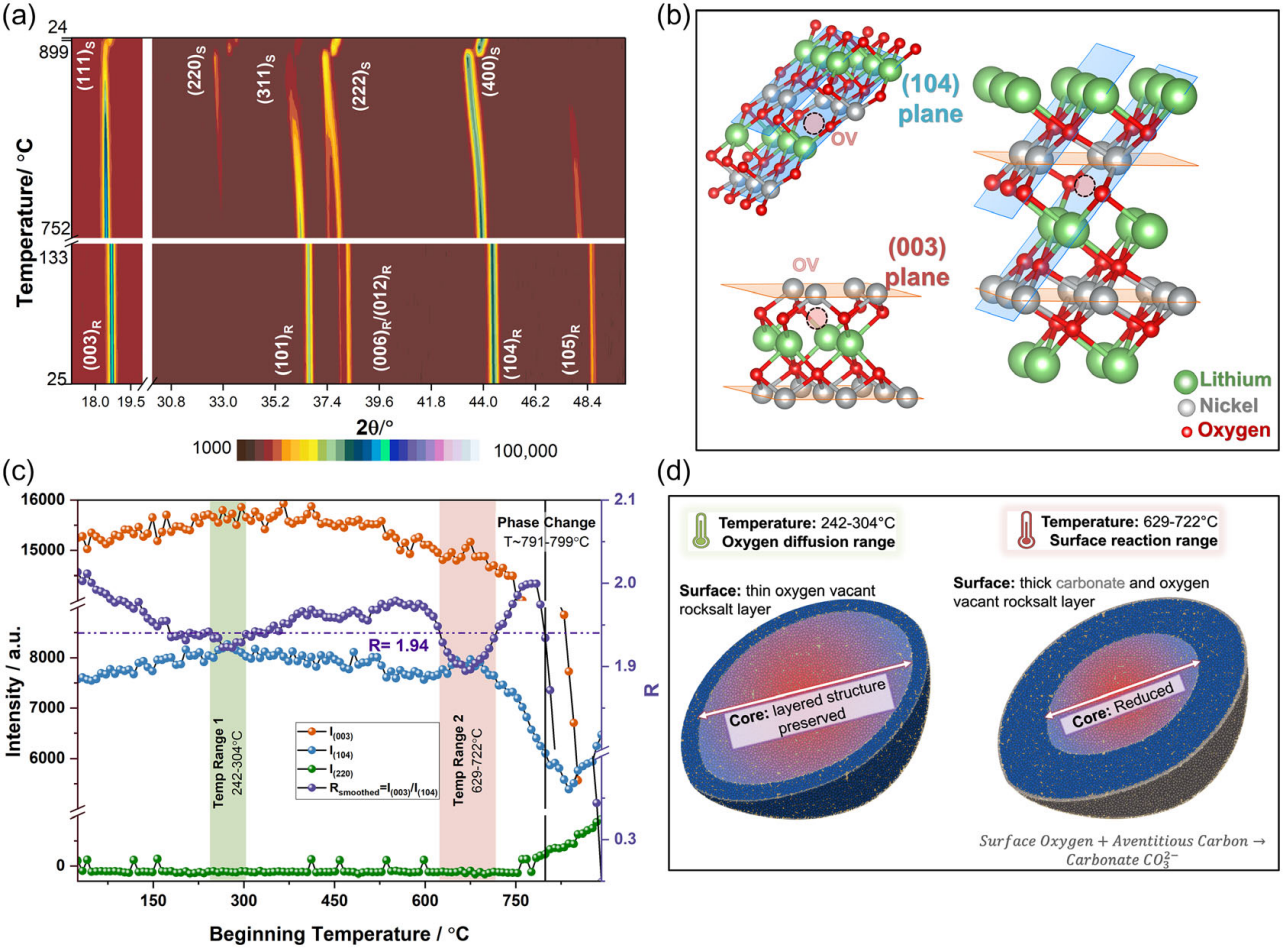
a) VT‐XRD analysis to identify optimum temperature window for surface OV formation. VT‐XRD patterns of NMC811 powder under nitrogen atmospheres annealed to 900 °C. A phase transformation to a spinel structure is observed at ≈791–799 °C, as indicated by the emergence of the (220) reflection. b) Schematic illustration of the (003) plane (orange) and (104) plane (blue) within the R$\bar{3}$m layered structure of LiNiO_2_. (003) plane intersects Li and Ni layer and can provide information regarding Li/Ni mixing. (104) plane intersects O alongside Li, Ni; OV homogenizes the plane, increasing the intensity. Hence, (104) serves as an indicator of OV formation. c) Temperature‐dependent intensity of the (003), (104), and (220) planes under nitrogen atmosphere. The ratio *R* = *I*_(003)_/*I*_(104)_ is used to identify two temperature windows (242–304 °C and 629–722 °C) that enable surface OV formation without triggering bulk phase transformation, as evidenced by the absence of the (220) reflection. d) Schematic representation of core–shell structure formation in different temperature ranges. At the lower temperature range oxygen leaves the structure by diffusion, whereas at a higher temperature oxygen leaves the surface and reacts with adventitious carbon to form surface carbonates.

VT‐XRD was conducted under air (Figure S2, Supporting Information) and nitrogen (**Figure** **3**a) atmospheres from room temperature to 900 °C. Under air, the layered R$\bar{3}$m structure remained intact throughout heating, with thermal expansion causing peak shifts to lower 2*θ* values. In contrast, under nitrogen, a spinel Ni_3_O_4_ (Fd$\bar{3}$m) phase emerged between 791 and 799 °C, marked by the appearance of the (220) reflection (Figure S3, Supporting Information),^[^ ^23^ ^]^ indicating oxygen loss and irreversible bulk phase transformation. The simulated XRD pattern for R$\bar{3}$m, Fd$\bar{3}$m, and Fm$\bar{3}$m is represented in Figure S4–S6, Supporting Information, respectively.

However, to preserve electrochemical performance, the phase transition must remain confined to the surface of the particles. Thus, the optimal temperature for inducing surface reconstruction must lie below the bulk layered‐to‐spinel transition threshold. At such intermediate temperatures, where only a small fraction of the particle volume undergoes transformation, the phase change cannot be directly resolved by XRD.

Notably, OV formation is closely coupled to the surface transition from the layered structure to rock‐salt phase.^[^ ^10^ ^]^ By analyzing changes in specific planar reflection intensities in XRD, we can approximate the extent of OV formation and, by extension, the degree of surface reconstruction. A closer observation of the crystal structure of layered phase (R$\bar{3}$m) in Figure 3b, illustrates how comparing different planes and their intensity might provide a reliable way of tracking OV formation. Namely, the (003) plane intersects Li^+^ or the transition metal, avoiding the planes with oxygen species, hence, the planar intensity *I*_(003)_ can be used as a normalization parameter. Furthermore, increase in cation disorder (Li^+^/Ni^2+^) can reduce the *I*_(003)_.^[^ ^24^ ^]^ Whereas the (104) plane intersects all the different species: O, Li, TM. The formation of OV reduces the variation across the plane and hence increases the planar intensity, *I*_(104)_. Hence, to probe surface changes, we tracked the *I*_(003)_/*I*_(104)_ peak intensity ratio (*R*).^[^ ^23^ ^]^ A decrease in *R*, prior to reaching the phase transformation temperature, indicate OV formation and/or cation disorder without bulk transformation. For the pristine NMC811 (25–33 °C) the *I*_(003)_/*I*_(104)_ in the same XRD equipment was 2.01. Two optimal windows were identified where *R* is lowest (*R *< 1.94): 242–304 °C and 629–722 °C (Figure 3c) indicating probable higher OV formation.

### 2.3 Effect of Prolonged Heating

To assess the effect of temperature with higher resolution, ex situ XRD was used to assess structural changes in NMC811 powders annealed under N_2_ at various temperatures (260–650 °C for 3 h). Figure S7, Supporting Information shows diffraction patterns for pristine (P_NMC811) and annealed samples (OV260_NMC811, OV280_NMC811, OV300_NMC811, OV630_NMC811, and OV650_NMC811 annealed at 260°, 280°, 300°, 630°, and 680 °C, respectively), the peak fitting with residual analysis is plotted in Figure S8, Supporting Information. All retained the layered R$\bar{3}$m structure, but OV300_NMC811 showed the sharpest (006)/(012) peaks (Figure S7c, Supporting Information), indicating improved long‐range ordering and fewer stacking faults.^[^ ^25^ ^]^

**Figure 4:**
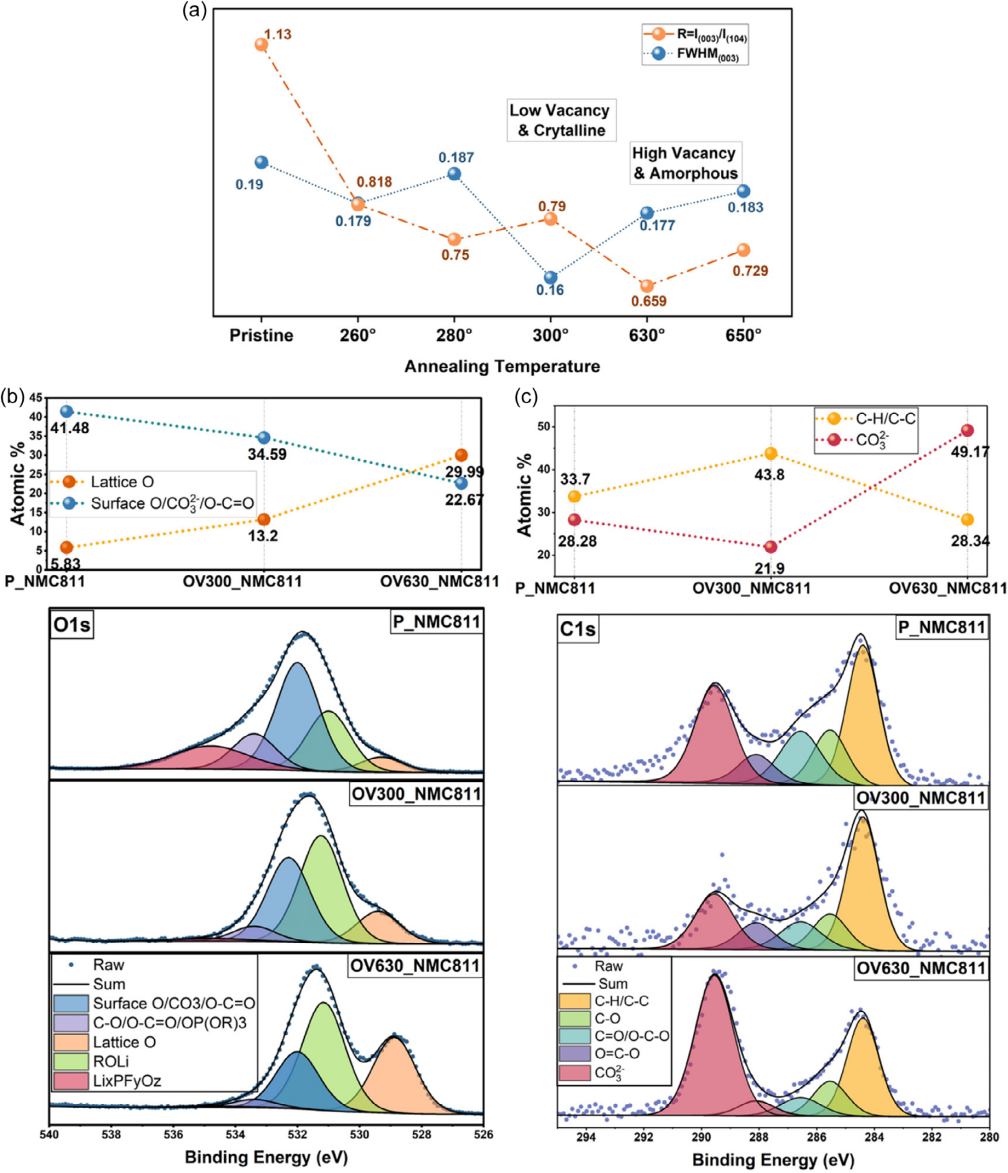
Temperature‐dependent structural evolution during prolonged annealing. a) Ex situ XRD: *R*‐value (*I*_(003)_/*I*_(104)_) and FWHM of (003) plane for NMC811 powders annealed under N_2_ atmosphere at various temperatures (260–650 °C) for 3 h. OV300_NMC811 exhibits the lowest FWHM with *R*‐value close to P_NMC811, suggesting minimal Li/Ni disorder, while OV630_NMC811 displays the lowest *R*‐value, indicating substantial OV formation and lattice disruption. These two conditions were selected as representative cases for further structural and spectroscopic analysis. b) Ex situ XPS‐O 1s of P_NMC811, OV300_NMC811, and OV630_NMC811 were deconvoluted into lattice O, surface O (CO_3_^2−^/C—O), and other surface species. Quantification reveals OV630_NMC811 has the lowest surface oxygen and highest lattice O signal, indicating significant OV formation. c) Ex situ XPS‐C 1s spectra shows carbonate and organic species. OV630_NMC811 exhibits the highest carbonate content (49.17%), suggesting that oxygen loss from the surface leads to reactive carbonate formation.

OV300_NMC811 had an *R*‐value closest to pristine, implying minimal Li/Ni mixing and limited OV content (**Figure** **4**a). In contrast, OV630_NMC811 showed the lowest *R*, suggesting significant OV generation and cation disorder. Analysis of the (003) peak's full width at half maxima (FWHM) revealed that all annealed samples had improved crystallinity compared to pristine, with OV300_NMC811 showing the sharpest peak and least disorder. OV630_NMC811, while richest in OVs, showed greater structural disruption. Based on this, OV300_NMC811 and OV630_NMC811 were selected as representative samples for further analysis, capturing the trade‐off between preserving crystallinity and maximizing OV content.

Furthermore, ex situ XRD analysis of the pristine and annealed powders (Figure S8, Supporting Information), stored in ambient atmosphere for 10 months, shows that all annealed samples maintain a lower *R*‐value compared to the pristine case. This trend might demonstrate the long‐term stability of OV and surface modification.

### 2.4 Reducing Surface Contamination

Bulk XRD data narrowed optimal annealing to two conditions: OV300_NMC811 and OV630_NMC811, but XRD cannot resolve surface‐localized chemical phenomena. To directly assess surface changes, XPS of P_NMC811, OV300_NMC811, and OV630_NMC811 samples were analyzed.

Figure 4b shows the O 1s spectra, deconvoluted (Table S2, Supporting Information) into lattice oxygen, residual Li carbonate (ROLi), surface species (e.g., CO_3_^2−^/O—C=O), organics, and fluorinated residues.^[^ ^26^ ^]^ Surface oxygen content, calculated from the surface O/CO_3_^2−^/O—C=O signal, decreased with annealing temperature. OV630_NMC811 showed the greatest loss (≈18.8%), while OV300_NMC811 experienced a moderate decrease (≈6.9%) indicating lower amount of OV formation and/or surface cleaning. Due to spectral overlap between the surface O and CO_3_^2−^/O—C=O signals, we also examined lattice oxygen signal that stands separate from other peaks. Notably, the lattice oxygen peak gets revealed^[^ ^27^ ^]^ as the surface is either etched or in our case undergoes surface oxygen removal. Both modified samples showed increased lattice oxygen percentages, consistent with surface oxygen depletion, with OV630_NMC811 again exhibiting the larger shift.

Furthermore, the deconvoluted (Table S3, Supporting Information) C 1s (Figure 4c) spectra were used to quantify CO_3_^2−^ species, as the formation of carbonates on the surface can significantly impact performance due to their poor conductivity.^[^ ^28^ ^]^ OV630_NMC811 showed excessive carbonate accumulation (49.2%) alongside a decrease in adventitious C—C/C—H, suggesting that oxygen at high temperatures react with surface carbon to form parasitic carbonates. In contrast, OV300_NMC811 had the lowest carbonate content (21.9%), even below pristine levels. This mechanism is illustrated in Figure 3d, where in the “oxygen diffusion range,” O diffuses out of the surface without reacting with surface C. Whereas, in the “surface reaction range,” surface O and adventitious C reacts to form CO_3_^2−^. Furthermore, the modified surface is predicted to be thicker at higher temperature due to a high concentration of OV alongside the loss of crystallinity in OV630_NMC811 and OV650_NMC811 (as seen in Figure 4a).

Furthermore, the deconvoluted (Table S3, Supporting Information) C1s (Figure 4c) spectra were used to quantify CO_3_^2−^ species, as the formation of carbonates on the surface can significantly impact performance due to their poor conductivity.^[^ ^28^ ^]^ OV630_NMC811 showed excessive carbonate accumulation (49.2%) alongside a decrease in adventitious C—C/C—H, suggesting that oxygen at high temperatures react with surface carbon to form parasitic carbonates. In contrast, OV300_NMC811 had the lowest carbonate content (21.9%), even below pristine levels. This mechanism is illustrated in Figure 3d, where in the “oxygen diffusion range,” O diffuses out of the surface without reacting with surface C. Whereas, in the “surface reaction range,” surface O and adventitious C reacts to form CO_3_^2−^. Furthermore, the modified surface is predicted to be thicker at higher temperature due to a high concentration of OV alongside the loss of crystallinity in OV630_NMC811 and OV650_NMC811 (as seen in Figure 4a).

In summary, OV630_NMC811 demonstrates high OV concentration but suffers from carbonate build‐up. In contrast, OV300_NMC811 achieves moderate OV formation while maintaining structural integrity. This contrast underscores a key design insight: beyond a certain threshold, vacancy formation triggers surface contaminant formation.

### 2.5 Surface Thickness and Phase Identification

Ex situ XPS shows increased Ni^2+^ content at the surface of annealed samples (Figure S9, Supporting Information), indicating the formation of a spinel or rock‐salt phase, while XRD (Figure S2, Supporting Information) confirms that the bulk retains the layered R‐3m structure, supporting the formation of a core–shell architecture.

**Figure 5:**
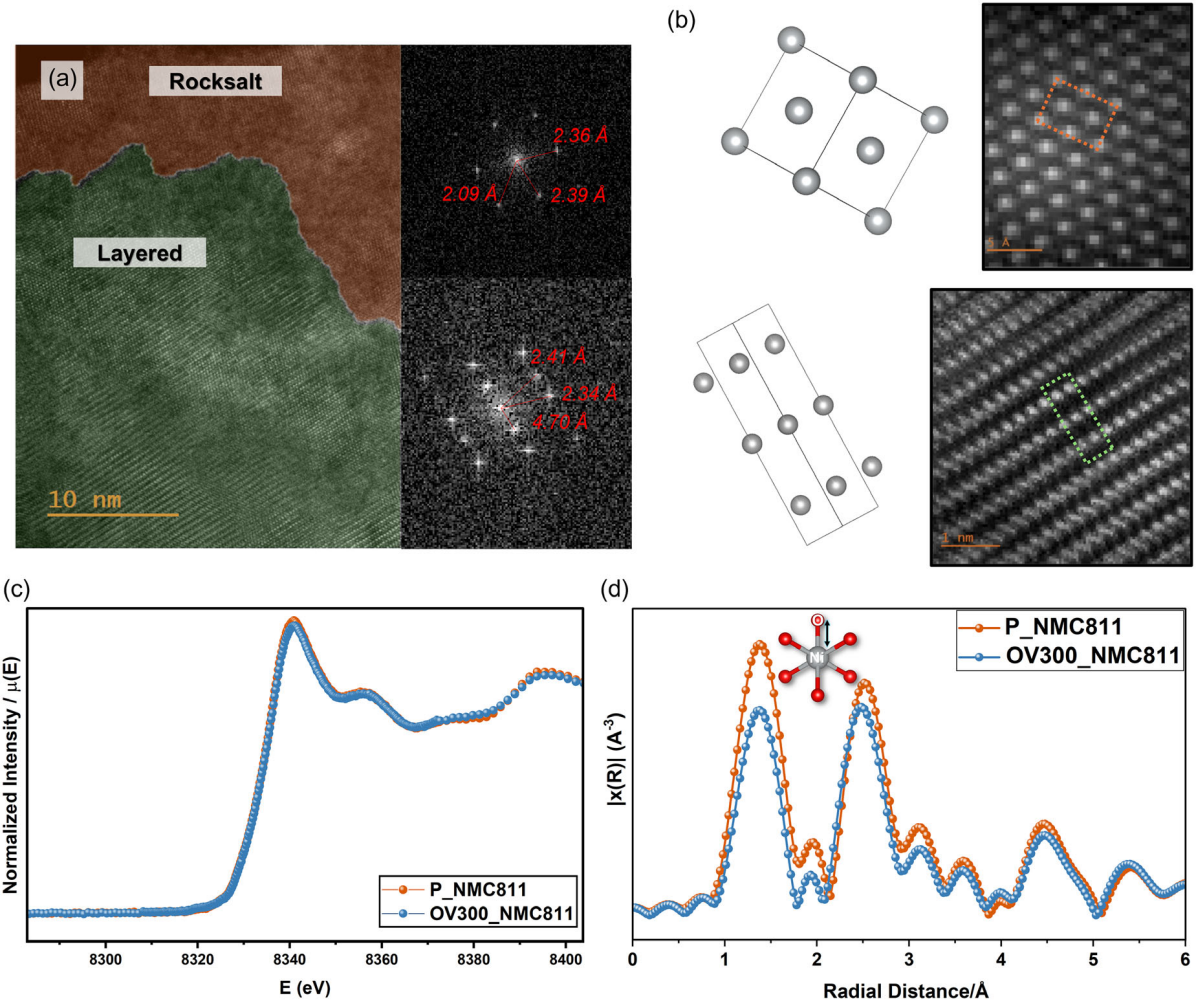
a) STEM Image of OV300_NMC811 displays the surface‐to‐core structural transformation, highlighting the engineered layer at the surface. An average of 10–20 nm shell with rock‐salt structure, and a homogenous layered core structure was observed in a 15 μm particle. b) Simulated atomic model overlaid on STEM image to identify the unit cell and confirm the structural match. c) h‐XAS of Ni K‐edge: Comparison of the Ni K‐edge h‐XAS spectra for P_NMC811 (orange) and OV300_NMC811 (blue). d) EXAFS R‐space analysis showing the FWHM for P_NMC811 (orange) and OV300_NMC811 (blue), highlighting changes in local atomic structure around nickel.

STEM imaging of OV300_NMC811 (**Figure** **5**a) reveals a ≈10–15 nm rock‐salt surface layer and a well‐preserved layered core, where the zone axis is <110> for both phases (Figure S10, Supporting Information). In contrast, P_NMC811 (Figure S10b, Supporting Information) shows a ≈40 nm mixed‐phase region, consistent with disorder from high‐temperature synthesis.^[^ ^7^, ^29^ ^]^ A structural agreement between the experimentally observed rock‐salt and layered interface and the simulated structure is seen in Figure 5b.

OVs, highlighted in Figure S11a, Supporting Information, coincide with surface edge dislocations, confirmed by histogram analysis. In Figure S12, Supporting Information, STEM‐energy‐dispersive X‐ray spectroscopy (EDS) mapping rule out the focused ion beam artifacts (Ga, Au, C) on surface, confirming that the observed surface reconstruction arises from annealing alone.

### 2.6 Oxidation State

XAS have been used extensively for identification of oxidation state in cathode materials.^[^ ^30^, ^31^, ^32^, ^33^, ^34^, ^35^, ^36^ ^]^ Figure 5c shows h‐XAS of the Ni K‐edge for P_NMC811 and OV300_NMC811. No significant shift is observed in our Ni K‐edge upon modification in the derivative plot (Figure S13a, Supporting Information), indicating that the bulk‐averaged oxidation state is preserved. Despite the homogenization effect observed in Figure 5a, where the bulk structure becomes predominantly layered compared to the more heterogeneous pristine sample (Figure S11b, Supporting Information), the oxidation state remains unchanged. Hence, the structural change is architectural: in OV300_NMC811, the rock‐salt phase is localized at the surface, whereas in the pristine sample, rock‐salt regions are more randomly distributed throughout the structure.

Fourier transformed extended X‐ray absorption fine structure (EXAFS) R‐space plot in Figure 5d shows no significant change in the average Ni‐O bond distance between OV300_NMC811 and P_NMC811. However, the OV300_NMC811 sample exhibits lower intensity. (Figure S13b, Supporting Information includes the associated K‐space). Since Figure 5c shows that both materials share the same oxidation state, this reduced intensity likely reflects local structural distortion.

### 2.7 Electrochemical Performance

**Figure 6:**
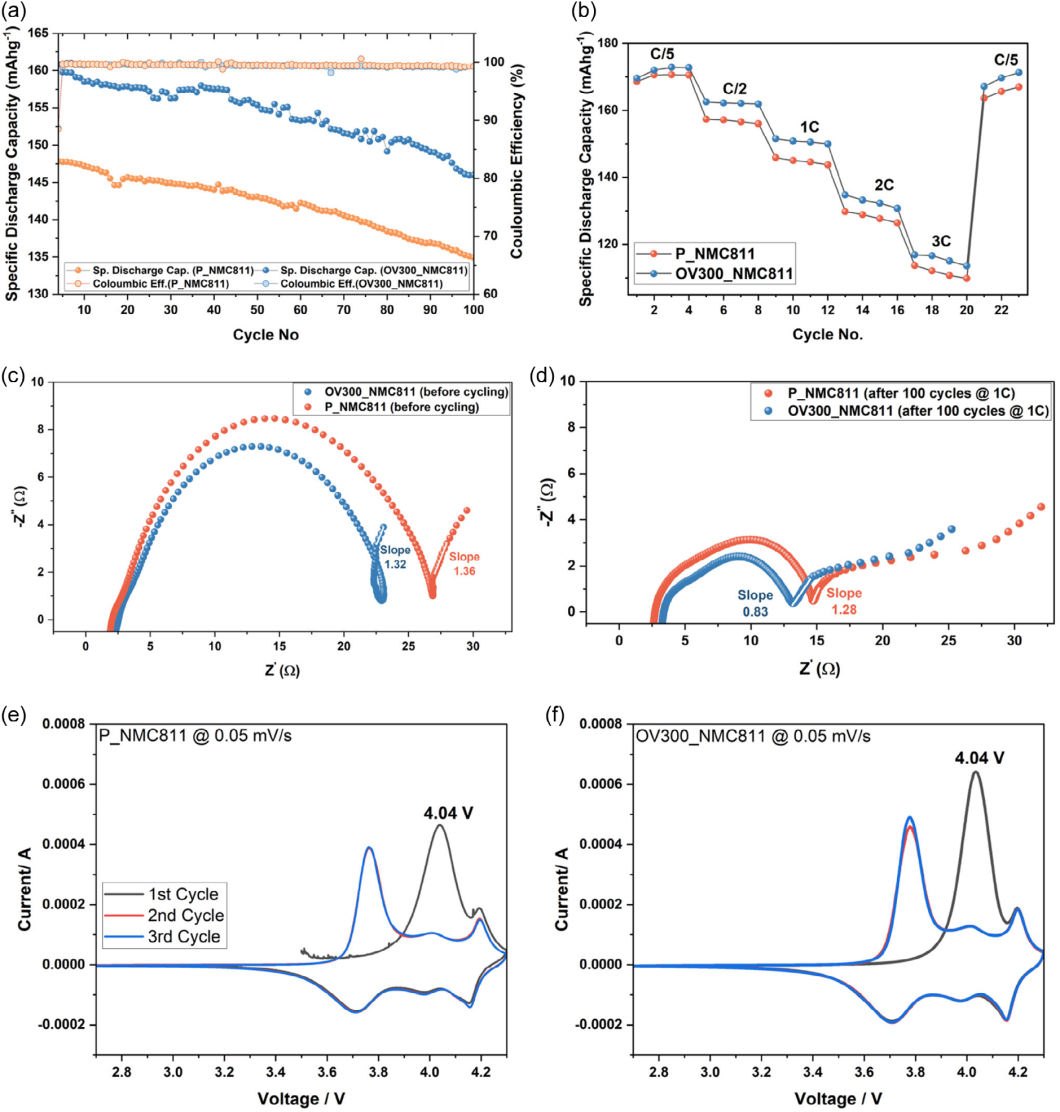
Electrochemical performance comparison of P_NMC811 and OV300_NMC811. a) Long‐term cycling performance at 1C after four formation cycles at C/10, showing improved capacity retention for OV300_NMC811. b) Rate capability from C/5 to 3C, demonstrating consistently higher capacities for OV300_NMC811. c,d) Nyquist plots before cycling and after 100 cycles, indicating lower resistance and improved Li^+^ diffusion in OV300_NMC811. e,f) Cyclic voltammetry profiles showing similar activation behavior for both cathodes.

**Figure** **6**a compares the long‐term cycling performance of P_NMC811 and OV300_NMC811 cathodes. Both cells underwent four formation cycles at C/10 (Figure S14, Supporting Information), followed by 100 cycles at 1C. OV300_NMC811 consistently delivered a higher discharge capacity throughout cycling. Notably, the 100th‐cycle capacity of OV300_NMC811 matched the first‐cycle capacity of P_NMC811, underscoring the enhanced structural and electrochemical stability imparted by the surface modification.

To further evaluate rate capability, C‐rate testing was performed for four cycles each at C/5, C/2, 1C, 2C, 3C, and back to C/5 (Figure 6b). OV300_NMC811 outperformed P_NMC811 at all rates, suggesting that OV‐induced homogenization as suggested from the STEM image (Figure 5a) improved Li^+^‐ion transport kinetics throughout the cathode.

Electrochemical impedance spectroscopy (EIS) measurements before cycling and after 100 cycles (Figure 6c,d) revealed that OV300_NMC811 cathodes exhibit consistently lower charge‐transfer resistance, despite both cells showing similar contact resistance. This reduction in impedance aligns with the earlier hypothesis that thermal treatment not only forms a NiO surface but also homogenizes the bulk, reducing intergranular heterogeneity that typically impedes Li^+^‐ion transport in Ni‐rich cathodes. While both samples showed comparable Warburg slopes before cycling, the postcycling slope of P_NMC811 became steeper, indicating slower Li‐ion diffusion, whereas OV300_NMC811 retained a more gradual slope, consistent with improved Li^+^ diffusivity.

Furthermore, a negative capacitance loop was observed at low frequency^[^ ^37^, ^38^ ^]^ for OV300_NMC811 before cycling, which was tested for reproducibility (Figure S15, Supporting Information) and disappeared after 100 cycles. This feature is characteristic of surface relaxation or oxide formation on the surface of electrochemical systems.^[^ ^38^ ^]^ Hence, we can attribute this loop to direct evidence of NiO on the surface. Its disappearance after extended cycling suggests the formation of a stable, electronically passivated interface that resists further reconstruction.

Cyclic voltammetry (Figure 6e,f) revealed identical activation energies for both P_NMC811 and OV300_NMC811, even though the surface of OV300_NMC811 contains NiO, a compound traditionally attributed to higher resistance.^[^ ^39^ ^]^ This confirms controlled thin NiO layer on cathode surface improved kinetics and stability.

### 2.8 Postcycling Characterization

**Figure 7:**
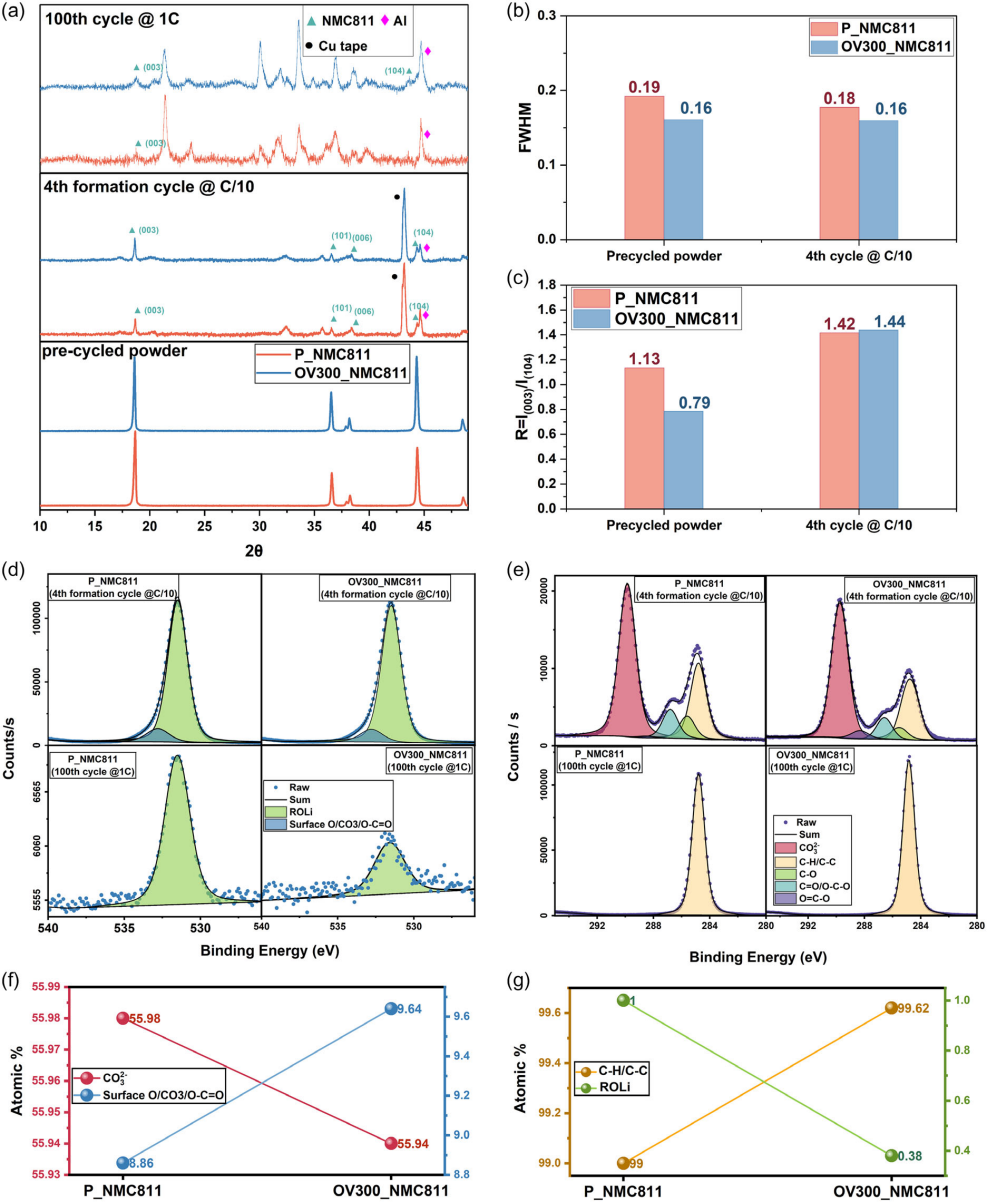
Postcycling structural and surface characterization of P_NMC811 and OV300_NMC811. a) XRD patterns of precycled, 4th‐cycle (C/10), and 100th‐cycle (1C) samples, showing stronger (003) and (104) reflections for OV300_NMC811. b,c) FWHM and *R*‐value comparing the precycled samples and the 4th‐cylce (C/10) sample. d,e) XPS‐O 1s and C 1s spectra after formation and 100th cycle respectively. f) Surface oxygen (from O 1s) and carbonate atomic% after formation cycle. g) Adventitious carbon and oxygen atomic% after 100th cycles.

To examine the structural evolution of P_NMC811 and OV300_NMC811 during electrochemical cycling, ex situ XRD was performed on the pristine powders, after the 4th formation cycle (C/10), and following the 100th cycle at 1C (**Figure** **7**a). In all cycled samples, additional reflections corresponding to Li_2_O, Li_2_CO_3_, and LiOH.H_2_O‐related phases were detected (Figure S16, Supporting Information), indicative of electrolyte decomposition and surface reconstruction upon extended cycling. These degradation products became more prominent after 100 cycles, consistent with increased interfacial reactions for extended cycling at higher C‐rate.^[^ ^40^, ^41^ ^]^ While the reflection for R$\bar{3}$m phases reduce after 100th cycle.

Despite these similarities, the OV300_NMC811 retained stronger (003) and (104) reflections across all cycling stages compared to the pristine counterpart, evidencing improved structural stability of the layered lattice. In contrast, the P_NMC811 sample exhibited severe decrease in intensity of NMC811 peaks after 100 cycles, suggesting extensive cation mixing and structural collapse, while OV300_NMC811 preserved its layered order.

The FWHM of the (003) plane of OV300_NMC811 sample remains consistent and lower compared to P_NMC811 during precycled and formation cycles (Figure 7b). In Figure 7c, the *R*‐value is lower in OV300_NMC811 on precycled powders, while the *R*‐value increase after formation cycle. A low *R*‐value indicates OV formation before cycling. However, the decrease in *R*‐value during cycling mostly corresponds to cation mixing as no additional process has been employed to induce additional vacancy formation. Hence, the P_NMC811 sample shows higher cation mixing compared to OV300_NMC811, which results in the lower intensity of (003) and (104) peaks after the 100th cycle at 1C (Figure 7a).

The surface chemistry evolution was further analyzed by XPS of the O 1s and C 1s regions for both materials after the 4th formation cycle and after 100 cycles at 1C (Figure 7d,e). During the formation cycle P_NMC811 has higher carbonate% compared to OV300_NMC811 whereas the surface O% is higher in OV300_NMC811 (Figure 7f), indicating in OV300_NMC811, surface oxygen reacts less to form carbonate species.

After 100 cycles, carbonate species and surface O component are no longer visible in C 1s and O 1s spectra. However, for comparable C 1s intensities, the O 1s signal was markedly lower in OV300_NMC811, corresponding to an overall oxygen content of 0.38% compared to 1% atomic oxygen for P_NMC811 (Figure 7g). This decrease is consistent with the presence of OV introduced by surface treatment, which likely act as stabilizing sites that suppress further oxygen loss during cycling.

After extended cycling, the Ni 2p spectra (Figure S17, Supporting Information) of both samples exhibited significant noise, indicating surface amorphization and the accumulation of insulating species. Nonetheless, after formation cycling, the Ni 2p signal of OV300_NMC811 was notably more pronounced with a FWHM of 87.77 compared to 149.22 of P_NMC811 under identical scan conditions, implying that the modified surface maintained less severe surface coverage by resistive layers, also seen from the reduced carbonate compound present in OV300_NMC811.

Together, these postcycling characterizations confirm that OV‐engineered cathodes sustain their structural framework and mitigate interfacial degradation during prolonged cycling, in agreement with the improved electrochemical performance observed in Figure 6.

## 3 Conclusion

Ni‐rich layered cathodes are among the most promising candidates for Li‐ion batteries but suffer from surface‐specific instabilities that accelerate degradation. In this work, we leverage one of the degradation pathways: surface reconstruction, to passivate itself. While NiO is widely regarded as an indicator of degradation in cathode, it is also a well‐studied, functional material in other electrochemical applications. As a wide‐bandgap, antiferromagnetic oxide and one of the few p‐type semiconductors among transition metal oxides, NiO exhibits tunable electronic properties^[^ ^42^, ^43^ ^]^ with broad applications in catalysis, gas sensing, and optoelectronics.^[^ ^44^, ^45^, ^46^, ^47^ ^]^ By harnessing these properties in battery, we challenge the conventional view of NiO as merely detrimental.

DFT calculations reveal that in NiO, the O 2p band center shifts further away from Fermi energy, weakening the covalent interaction with Ni 3d and enhances stability. Notably, the surface ligand p‐band center is reported to be the most accurate DFT descriptor for measuring the hydrogen adsorption tendency on surface,^[^ ^20^ ^]^ which is essential for stable electrolyte–electrode interface. The higher energy ligand p‐band center of LNO makes it more susceptible to electrolytic attack, compared to NiO. Furthermore, surface phases with bandgaps can inhibit electrolyte oxidation,^[^ ^20^ ^]^ which is why using Al_2_O_3_ coatings^[^ ^1^, ^14^, ^22^, ^48^ ^]^ has shown improvement. NiO is a wide‐bandgap insulator that is inherent to Ni‐rich chemistry, hence leveraging the materials’ own chemistry should be explored.

However, DFT alone does not resolve the optimal processing conditions for NiO formation. In literature, the formation of NiO has been linked to areas with higher concentration of defects and OV.^[^ ^10^ ^]^ As the formation of OV can be measured by XRD analysis, we conducted a systematic VT‐XRD, that revealed two optimal thermal windows for surface OV formation (242–304 °C and 629–722 °C under N_2_ atmosphere). Interestingly, the surface OV formation temperature in literature often falls within the second temperature range.^[^ ^49^ ^]^ A common cause of using this high temperature in literature is reducing surface impurity. However, ex situ XPS analysis reveals a significant carbonate formation at high temperature and annealing at lower temperature yielded the best result.

STEM was used to identify the modified surface as rock‐salt phase and the bulk layered structure shows more homogeneity. Notably, we found a high density of dislocations on the modified surface. The overlap between dislocation and NiO phase confirmed the hypothesis that localized phase transformation can be triggered using OV generation. Interestingly, h‐XAS reveals that there is no difference in the bulk‐averaged oxidation state between the pristine and the modified cathode. However, the structure becomes more crystalline as evidenced by the FWHM of ex situ XRD.

OV300_NMC811 exhibits higher capacity and rate capability, maintaining higher Li^+^ diffusion and lower resistance after 100 cycles. The initial negative capacitance loop seen in EIS is a direct evidence of OV‐induced NiO layer, which vanishes with cycling as the interface stabilized, demonstrating that controlled surface reconstruction yields a durable, low‐resistance cathode architecture. Postcycling XRD shows OV‐engineered NMC811 retains distinct (003) and (104) peaks after 100 cycles, unlike the pristine sample, indicating better structural stability. XPS confirms lower O 1s intensity consistent with sustained OVs. These results highlight enhanced structural and surface stability enabled by controlled defect engineering.

The principles demonstrated here extend beyond NMC811. Since stabilization arises from tuning the O 2p–Ni 3 d interactions near the Fermi level, comparable effects are expected in other Ni‐rich layered oxides such as LiNiO_2_, NCA, and NMC622. Our hypothesis‐driven workflow (Figure 1d) integrating VT‐XRD with XPS‐guided surface analysis provides a generalizable route to identify optimal processing conditions for structural stabilization and performance enhancement.

Furthermore, while incomplete^[^ ^50^ ^]^ and complete^[^ ^51^ ^]^ surface reconstruction have been shown to have a surface passivation effect alongside cathode performance improvements, the temperature used for the modification method was higher than the ones used in the current work.

In summary, we introduce a universal and scalable strategy that derives stability from the intrinsic chemistry of the material itself. We demonstrate this principle in Ni‐rich cathodes, by repurposing surface NiO formation, typically regarded as a degradation marker, into a functional, self‐passivating layer. Rather than relying on extrinsic coatings or dopants,^[^ ^51^ ^]^ we enable this transformation through a single‐step thermal treatment that induces OV‐driven surface reconstruction. This process can be readily monitored using standard XRD technique and is inherently compatible with commercial‐scale manufacturing. Our study highlights the strength of combining theoretical predictions with rigorous experimental validation across multiple modalities (XRD, XPS, XAS, and STEM/EDS) to convert a known failure mode into a robust design principle. The resulting cathode materials exhibit improved long‐term electrochemical performance, demonstrating that surface reconstruction can be a powerful, materials‐intrinsic approach to next‐generation battery stability.

## Supporting Information

Supporting Information is available from the Wiley Online Library or from the author.

## Conflict of Interest

The authors declare no conflict of interest.

## Supporting information

Supplementary Material

## Data Availability

The data that support the findings of this study are available from the corresponding author upon reasonable request.
