# Supplementary material for: Surface Reconstruction as a Design Principle for Ni‐rich Cathodes
Source: Small Sci. 2025 Dec 10;6(1):e202500503. doi: 10.1002/smsc.202500503 (PMC12794674; doi:10.1002/smsc.202500503)
Supplement: Supplementary file 1 — Supplementary Material [file SMSC-6-e202500503-s001.pdf]

Supporting Information:

Surface reconstruction as a design principle for Ni-rich cathodes

Sumaiyatul Ahsan[b], Abiram Krishnan[b], Mengkun Tian[c], Samir Sarma[b] and Faisal M. Alamgir* [a]

Computational Method:

Density Functional Theory (DFT) calculations were performed on a unitcell of pristine $LiNiO_2$ (LNO) and NiO consisting of 12 and 8 atoms, respectively. The calculations were carried out using Quantum ESPRESSO[1,2], an open-source software package. All atoms were described using projector augmented wavefunctions (PAW), with a plane-wave cutoff of 103 Ry for the wavefunction. Recognizing the limitations of some functionals in the pseudopotentials for Ni atoms-specifically the potential for electron delocalization leading to inaccuracies in Coulombic interactions between d-orbital electrons and bandgap predictions, applied a Hubbard correction (U = 3) to Ni-3d for LNO[3]. However, due to NiO being a wide bandgap insulator, we used a U contribution of 9 for O-2p, which results in a wider bandgap[4], alongside 6.4 for Ni-3d with an interaction parameter J of 1 for both Ni-ed and O-2p.
Prior to density of states (DOS) calculations, both LNO and NiO structures underwent variable-cell relaxation. Structural optimization was performed to a self-consistency threshold of 1×10^{-6} to ensure convergence. All calculations were done under spin-polarized conditions to account for the antiferromagnetism and ferromagnetism of NiO and LNO respectively. NiO was relaxed with a uniform 3×3×3 k-mesh, and LNO was relaxed using a 3×3×1 k-mesh. Finally, a 6×6×6 and 9×9×9 k-mesh were used respectively for the self-consistent field (SCF) calculations and non-self-consistent field (NSCF) calculations of NiO with a 1×10^{-8} electronic convergence threshold. For LNO we used a 5×5×1 k-mesh for SCF calculations, and 10×10×2 k-mesh for NSCF calculations.

Characterization:

In-situ X-ray powder diffraction was performed using a Rigaku SmartLab XE instrument equipped with Cu $K\alpha_1$ radiation (1.540562 Å). The system was integrated with an Anton Paar HTK 2000N High-Temperature Chamber for atmosphere control. The sample was heated at 1°C/min and then rapidly cooled at 200°C/min. XRD data were collected with a scanning rate of 10°/min over a 2θ range of 15°-80°. For post-annealing analysis, XRD spectra were obtained using a Rigaku Miniflex instrument at the same scanning rate (10°/min) over a range of 15°-90° (2θ). X-ray photoelectron spectroscopy (XPS) was conducted on the annealed samples using the Thermo K-Alpha XPS system to determine the oxidation states of various elements.
For the preparation of thin cross-sections of both pristine and modified powders, Focused Ion Beam (FIB) milling was performed using the Thermo Fisher Helios 5CX. The final polishing condition is 43pA 5kV. Atomic resolution scanning transmission electron microscopy (STEM) images were captured using a Hitachi HD-2700, enabling precise phase identification and shell thickness measurements. The spatial resolution is 0.13 nm.

X-ray absorption measurements:

Transmission-mode measurements of nickel K-edge were collected using a lab-scale instrument which employs the Rowland circle geometry to achieve energy tunability of X-rays, produced by a bremsstrahlung source. Nickel K-edge spectra for pristine and modified samples were normalized using Athena from the Demeter software package[5]. The extended region was used to calculate interatomic distances using a Hanning window between 2.3 to 11 $Å^{-1}$ (K-space provided in **Figure S14b**).

STEM-EDS to investigate Surface damage due to FIB:

The surface of the electrode can get damaged during the FIB process that uses gallium (Ga) as the ion source. We used a carbon and gold coating on top of the cathode particle to reduce mechanical damage and disperse the heat generated during the process of extracting the particle cross-section. Hence, an

energy dispersive spectroscopy (EDS) was performed in conjunction with STEM using a 1-2 nm resolution to analyze the chemical composition of the surface to make sure the defect and phase transformation is a result of surface modification and not contamination.

Electrochemical Performance:

Pristine NMC811 powders were sourced from MSE Supplies, with an average particle size of 10.0 µm ± 2.0 µm. Electrodes were prepared by mixing the active material (NMC811), Polyvinylidene Fluoride (PVDF) binder, and high-purity conductive carbon black (Super C45) in an 8:1:1 weight ratio. The average active weights of the electrodes were 3.46 mg for pristine NMC811 and 3.24 mg for the modified NMC811, each covering an area of 1.27 cm^2. The total wet thickness of the electrodes was 85 µm. Electrochemical cycling of the CR2032[6] half-cells was performed at a constant current rate of 1C, following three formation cycles at a rate of C/10 (0.1C). The currents were calculated using a theoretical capacity of 275 $mAhg^{-1}$ for NMC811.

Table S1: Centroid shift calculation of partial density of states of Ni3d and O2p

**Ni3d**	**LNO**	**NiO**	**O2p**	**LNO**	**NiO**
Beginning energy	-8	-12	Beginning energy	-8	-11.9995
Ending energy	3	4.99886	Ending energy	3	4.99886
Max Height	12.14	19.08	Max Height	11.806	6.856
Area	29.57	39.5106	Area	32.565	21.94997
Centroid	-2.523	-2.7385	Centroid	-2.9702	-4.60913
FWHM	7.333	8.6029	FWHM	6.0462	6.75544

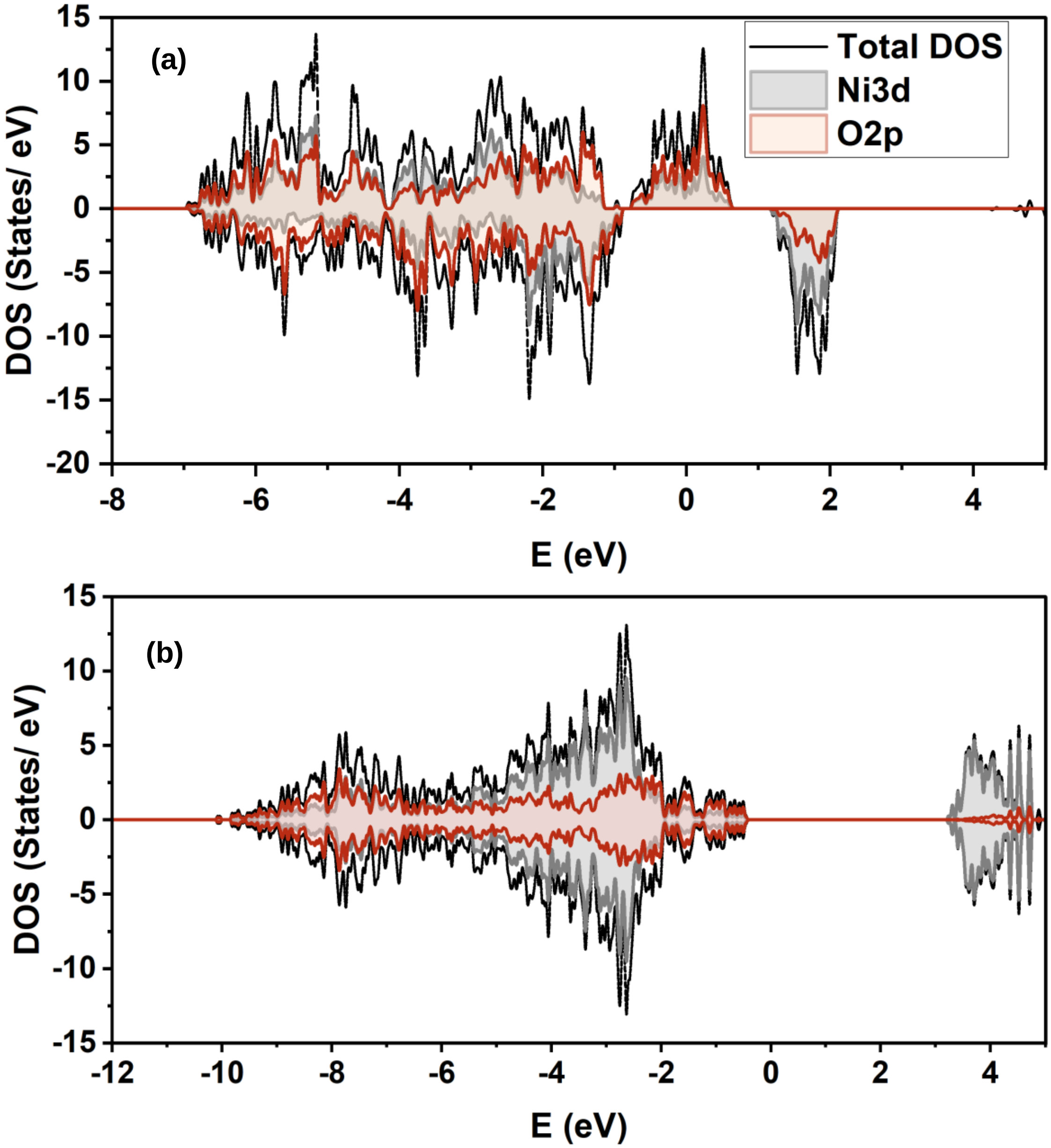

Figure S1. Spin-resolved density of states (DOS) for (a) $LiNiO_2$ (LNO) and (b) NiO. The DOS for LNO shows asymmetric spin-up and spin-down channels, indicating ferromagnetic metallic behavior with no bandgap. In contrast, NiO displays symmetric spin states and a clear bandgap, characteristic of an antiferromagnetic insulating phase. These distinctions underscore the electronic and magnetic transformation associated with the LNO to NiO surface reconstruction.

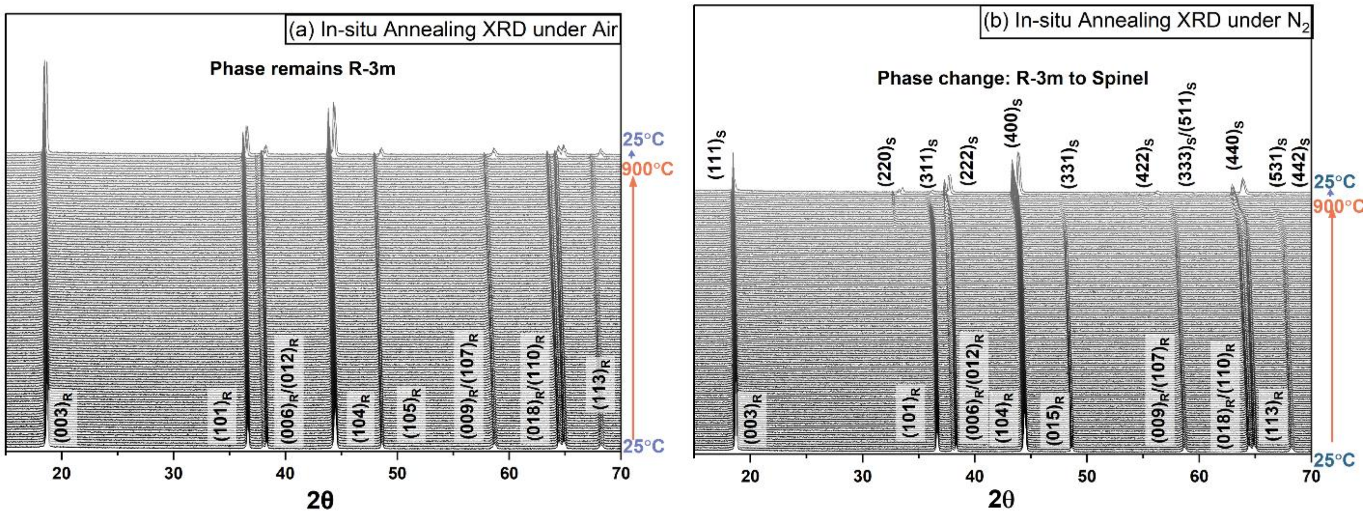

Figure S2. In situ annealing XRD of NMC811 under (a) air and (b) N_2 atmospheres. Under air, no significant phase transition is observed, indicating structural stability. In contrast, annealing under N_2 induces a clear phase transition from the layered structure to a spinel-like phase, demonstrating the atmosphere-dependent surface reconstruction behavior critical to controlled NiO formation.

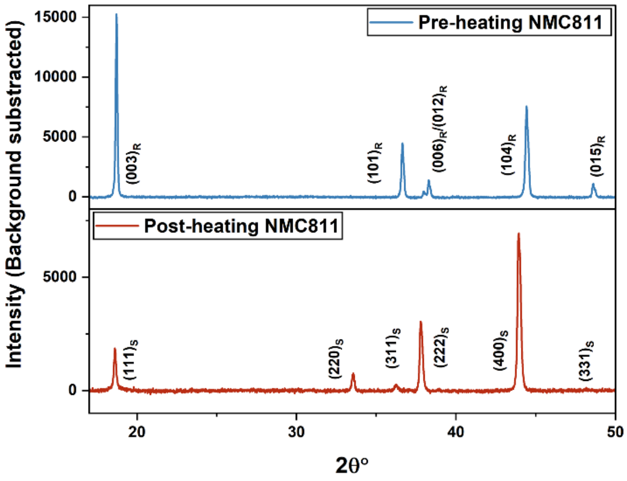

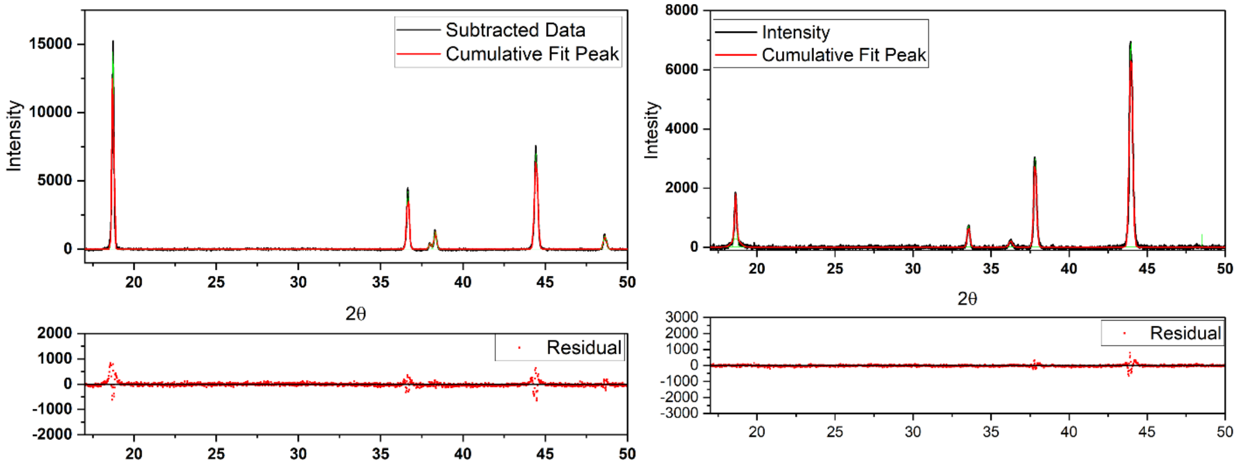

Figure S3. (a) XRD patterns of NMC811 before heating (24°C) and after annealing to 900 °C followed by cooling under N_2 (from Figure S2b).

The pre-heating sample shows a layered structure, while the post-heating sample exhibits a spinel phase that remains stable upon cooling, indicating an irreversible phase transformation.

(b) Peak fitting and residuals of the pre-heating XRD pattern confirm the layered R-3m symmetry.

(c) Peak fitting and residuals of the post-heating pattern are consistent with a spinel-type structure (Fd-3m), supporting the formation and stabilization of the reconstructed phase.

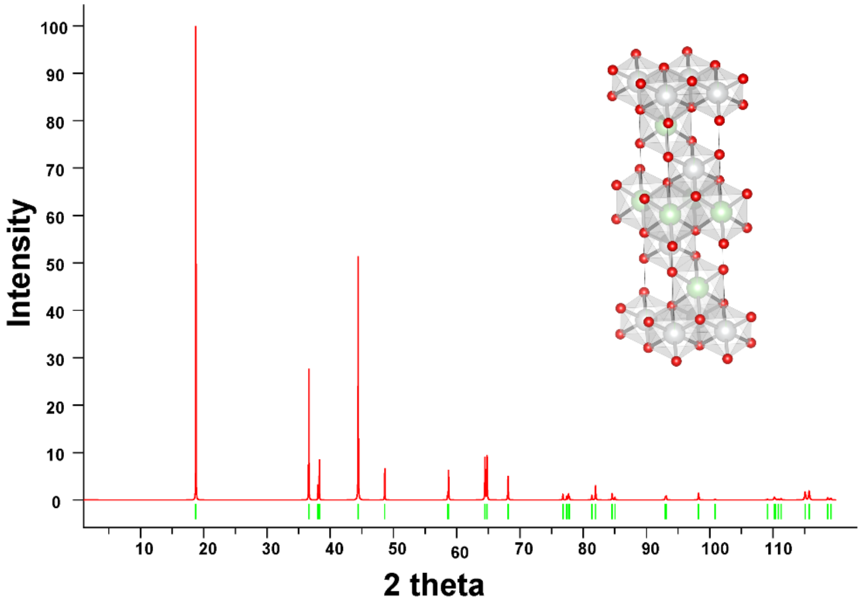

h	k	l	d (Å)	F(real)	F(imag)	|F|	2θ	I	M	ID(λ)	Phase
0	0	3	4.726853	65.05841	1.472749	65.0751	18.7577	100	2	1	1
1	0	1	2.452715	47.3174	1.431805	47.3391	36.60799	38.51357	6	1	1
0	0	6	2.363427	29.72116	1.327937	29.7508	38.04308	4.65857	2	1	1
0	1	2	2.349546	28.21966	1.319244	28.2505	38.27655	12.43239	6	1	1
1	0	4	2.037752	85.99928	1.698378	86.016	44.4213	82.65672	6	1	1
0	1	5	1.871271	35.92761	1.391327	35.9545	48.61623	11.77332	6	1	1
0	0	9	1.575618	27.85784	1.353156	27.8907	58.53469	1.54876	2	1	1
1	0	7	1.571485	47.19184	1.512299	47.2161	58.70366	13.22965	6	1	1
0	1	8	1.444092	65.84754	1.685463	65.8691	64.4726	20.92014	6	1	1
1	1	0	1.437745	67.51988	1.702749	67.5413	64.7919	21.7619	6	1	1
1	1	3	1.375523	38.07107	1.472749	38.0995	68.11196	12.45548	12	1	1
0	2	1	1.240351	30.75254	1.431805	30.7859	76.78289	3.22889	6	1	1
1	0	10	1.23227	25.00161	1.344927	25.0378	77.37962	2.1076	6	1	1
1	1	6	1.228319	23.34985	1.327937	23.3876	77.6751	3.6543	12	1	1
2	0	2	1.226358	22.50987	1.319244	22.5485	77.82272	1.693	6	1	1
0	0	12	1.181713	52.7028	1.66459	52.7291	81.36203	2.87839	2	1	1
0	2	4	1.174773	55.38786	1.698378	55.4139	81.94523	9.43969	6	1	1
0	1	11	1.144835	38.47189	1.548655	38.5031	84.57401	4.37085	6	1	1
2	0	5	1.14009	24.79249	1.391327	24.8315	85.00858	1.8067	6	1	1
1	1	9	1.062043	19.92656	1.353156	19.9725	92.98707	2.16182	12	1	1
0	2	7	1.060775	32.5136	1.512299	32.5488	93.13153	2.86848	6	1	1
2	0	8	1.018876	46.51295	1.685463	46.5435	98.23017	5.7831	6	1	1
1	0	13	0.99916	15.99345	1.319028	16.0477	100.8775	0.68953	6	1	1
0	0	15	0.945371	33.17322	1.580166	33.2108	109.1369	1.03533	2	1	1
2	1	1	0.939159	22.57803	1.431805	22.6234	110.2093	2.91378	12	1	1
0	1	14	0.938253	20.28438	1.369443	20.3306	110.368	1.17851	6	1	1
0	2	10	0.935636	18.5054	1.344927	18.5542	110.8304	0.98646	6	1	1
1	2	2	0.93304	16.65551	1.319244	16.7077	111.2941	1.60802	12	1	1
1	1	12	0.91292	39.51222	1.66459	39.5473	115.0812	9.45133	12	1	1
2	1	4	0.909709	41.62503	1.698378	41.6597	115.72	10.5835	12	1	1
2	0	11	0.895593	28.99756	1.548655	29.0389	118.6549	2.68977	6	1	1
1	2	5	0.893315	18.47603	1.391327	18.5283	119.1492	2.20797	12	1	1
2	1	7	0.85359	25.01717	1.512299	25.0628	128.9558	4.88931	12	1	1

Figure S4. Simulated XRD pattern of $LiNiO_2$ (LNO), showing all corresponding crystallographic peaks indexed to the R-3m layered structure. The simulation highlights the characteristic (003), (101), (104), (006/012), and (018) reflections, serving as reference for phase identification and comparison with experimental data.

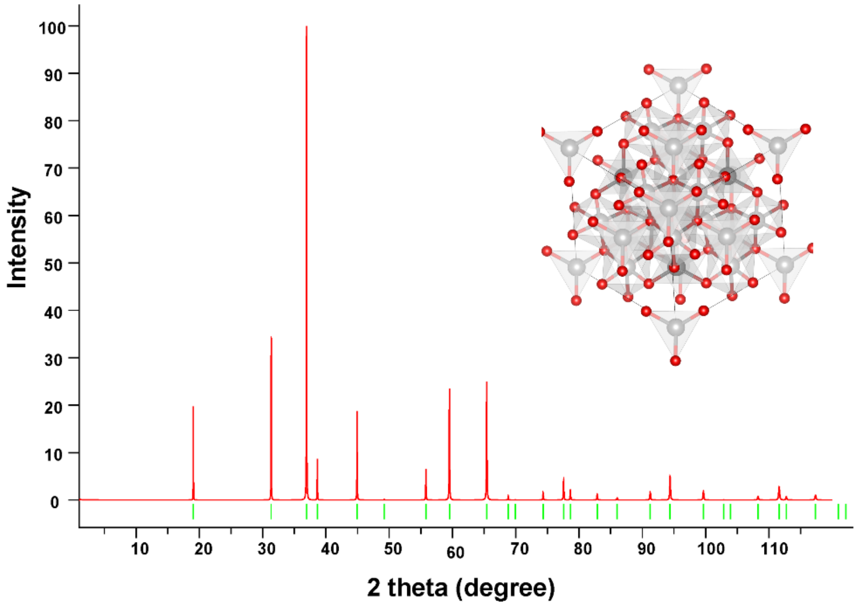

h	k	l	d (Å)	F(real)	F(imag)	|F|	2θ	I	M	ID(λ)	Phase
1	1	1	4.65633	-61.8547	49.66611	79.3267	19.04443	14.37432	8	1	1
2	2	0	2.851408	158.2895	28.90814	160.908	31.34591	31.12253	12	1	1
3	1	1	2.431689	134.1324	203.625	243.833	36.93592	100	24	1	1
2	2	2	2.328165	56.59713	-118.32	131.16	38.64196	8.72989	8	1	1
4	0	0	2.01625	-278.297	-29.6504	279.872	44.92072	21.29045	6	1	1
3	3	1	1.850238	-12.2385	0.659981	12.2563	49.20531	0.13285	24	1	1
4	2	2	1.646261	113.5916	28.4784	117.107	55.79699	9.11082	24	1	1
3	3	3	1.55211	71.19287	139.3411	156.475	59.50969	4.68802	8	1	1
5	1	1	1.55211	192.9037	-123.872	229.251	59.50969	30.18874	24	1	1
4	4	0	1.425704	412.303	85.84601	421.145	65.40704	41.37196	12	1	1
5	3	1	1.363234	-23.2803	-35.1404	42.1524	68.81165	1.48984	48	1	1
4	4	2	1.344167	-0.1012	10.44568	10.4462	69.92864	0.04427	24	1	1
6	2	0	1.275188	87.13607	28.05496	91.5411	74.32315	3.02029	24	1	1
5	3	3	1.229901	142.8307	-75.1084	161.375	77.55647	8.70463	24	1	1
6	2	2	1.215844	-55.2274	101.8958	115.9	78.62483	4.38943	24	1	1
4	4	4	1.164082	-159.765	-28.784	162.337	82.86234	2.65403	8	1	1
5	5	1	1.129326	-47.1892	35.07997	58.7999	86.01361	0.99745	24	1	1
7	1	1	1.129326	0.317795	-10.9483	10.9529	86.01361	0.03461	24	1	1
6	4	2	1.077731	84.75349	27.83416	89.207	91.24365	4.3533	48	1	1
5	5	3	1.049974	71.48558	138.6675	156.009	94.38386	6.53815	24	1	1
7	3	1	1.049974	108.1251	-41.7127	115.892	94.38386	7.21593	48	1	1
8	0	0	1.008125	276.6971	83.51575	289.026	99.6519	5.56844	6	1	1
7	3	3	0.985297	14.24387	3.490638	14.6653	102.8498	0.05785	24	1	1
6	4	4	0.978025	0.088137	-6.40263	6.40324	103.9244	0.01108	24	1	1
8	2	2	0.950469	69.05617	27.42047	74.301	108.2775	1.53931	24	1	1
6	6	0	0.950469	82.72883	27.61434	87.2159	108.2775	1.06047	12	1	1
5	5	5	0.931266	137.9185	-71.2047	155.215	111.6141	2.31693	8	1	1
7	5	1	0.931266	43.75236	109.6597	118.066	111.6141	8.04352	48	1	1
6	6	2	0.925119	53.88034	-79.6999	96.2038	112.743	2.70645	24	1	1
8	4	0	0.901694	-111.324	-27.9426	114.778	117.3598	4.10853	24	1	1
7	5	3	0.885249	-11.728	0.679054	11.7476	120.9514	0.09137	48	1	1
9	1	1	0.885249	-36.2292	24.44585	43.7053	120.9514	0.63233	24	1	1
8	4	2	0.879964	0.184262	-12.0418	12.0432	122.1783	0.09818	48	1	1
6	6	4	0.859732	46.16376	26.84175	53.4001	127.2674	1.06775	24	1	1

Figure S5. Simulated XRD pattern of Ni_3O_4 spinel phase, with all major peaks indexed to the Fd-3m space group. Characteristic reflections such as (111), (220), (311), and (400) confirm the spinel structure, providing a reference for identifying surface phase transitions in annealed samples.

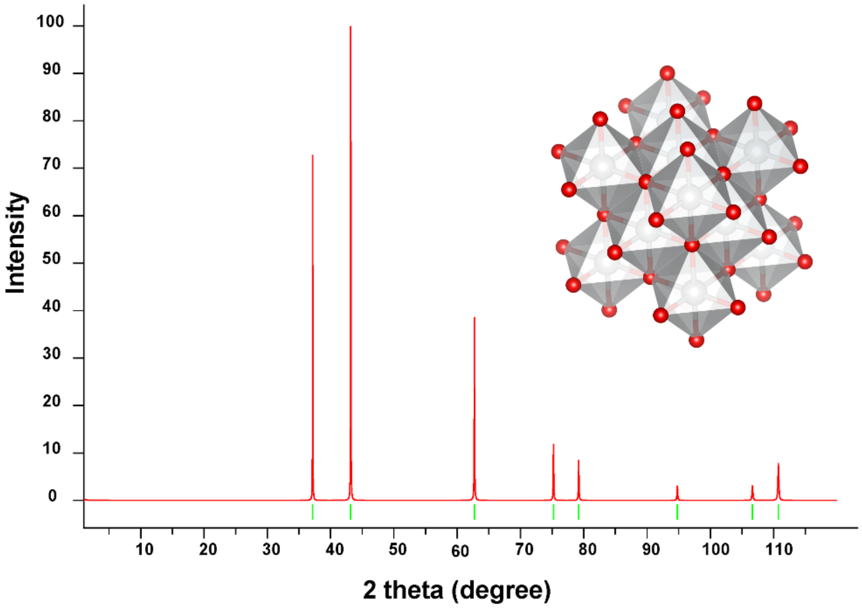

h	k	l	d (Å)	F(real)	F(imag)	|F|	2θ	I	M	ID(λ)	Phase
1	1	1	2.416983	52.78873	1.884393	52.8223	37.16885	63.21375	8	1	1
2	0	0	2.093169	90.6224	2.140794	90.6477	43.18526	100	6	1	1
2	2	0	1.480094	71.90611	2.140794	71.938	62.72322	54.17919	12	1	1
3	1	1	1.262228	37.75962	1.884393	37.8066	75.21765	20.65134	24	1	1
2	2	2	1.208492	59.83851	2.140794	59.8768	79.19692	15.81494	8	1	1
4	0	0	1.046584	51.36225	2.140794	51.4068	94.78524	7.26024	6	1	1
3	3	1	0.960412	28.21942	1.884393	28.2823	106.6523	9.01641	24	1	1
4	2	0	0.936094	45.0869	2.140794	45.1377	110.7491	23.85271	24	1	1
4	2	2	0.854533	40.273	2.140794	40.3299	128.6919	25.73513	24	1	1

Figure S6: Simulated XRD pattern of NiO rocksalt structure, indexed to the Fm-3m space group. The prominent peaks corresponding to the (111), (200), (220), (311), and (222) planes confirm the characteristic cubic symmetry, serving as a reference for identifying NiO formation in surface-reconstructed cathodes. The presence of (200) peak can differentiate between the spinel and rocksalt phase.

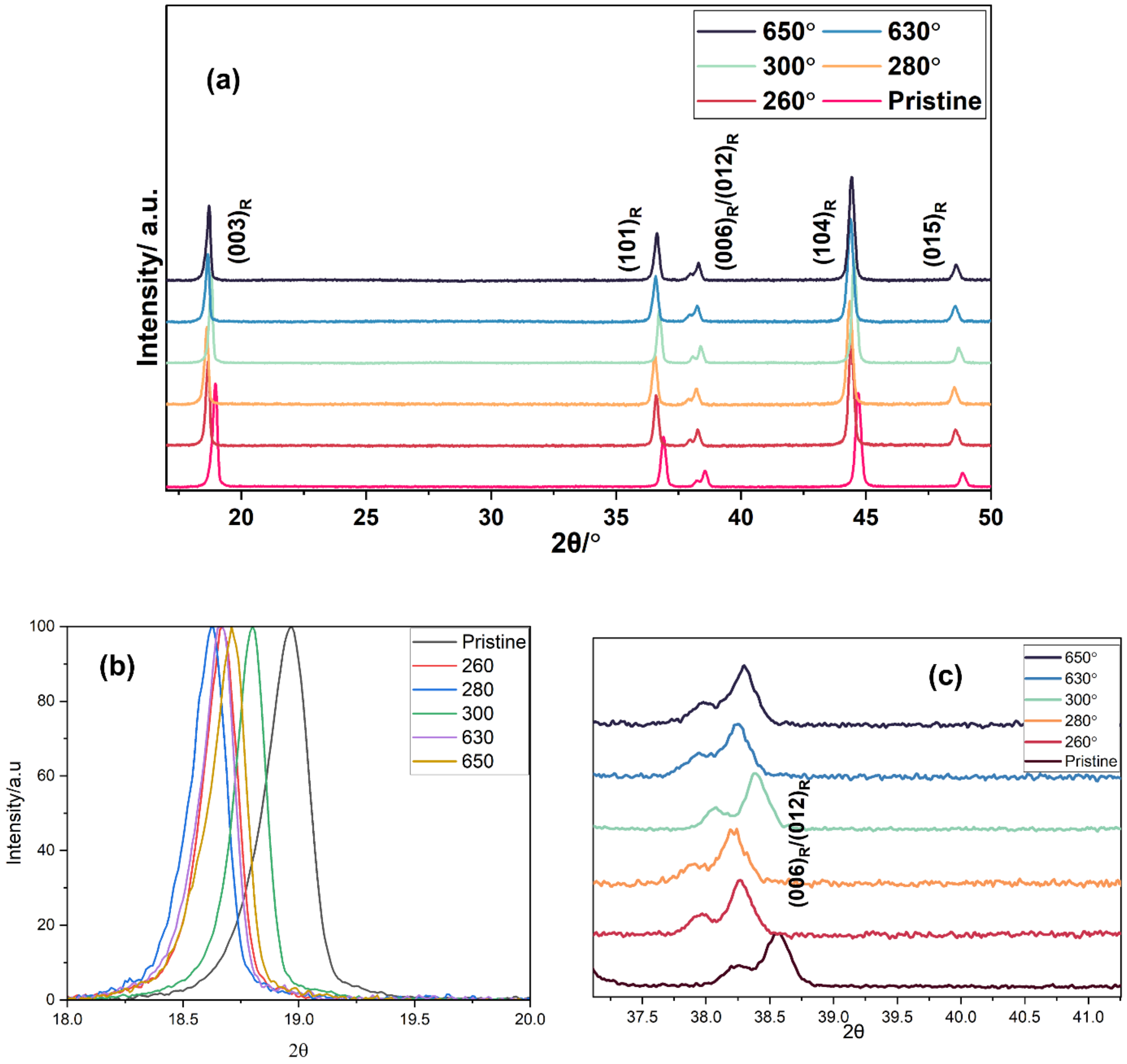

Figure S7.

(a) Ex situ XRD patterns of pristine NMC811 (P_NMC811) and modified samples annealed at 260°C, 280°C, 300°C, 63 °C, and 650°C (denoted as OV260_NMC811, OV280_NMC811, OV300_NMC811, OV630_NMC811 and OV650_NMC811) all showing retention of the layered R-3m structure.

(b) Zoomed-in view of the (003) reflection reveals peak shifts, indicative of subtle lattice changes with annealing.

(c) The (006)/(012) doublet shows improved peak separation and sharpness in the lower-temperature OV samples (260°C-30 °C), suggesting enhanced crystallinity and reduced structural disorder compared to pristine and high-temperature-treated samples.

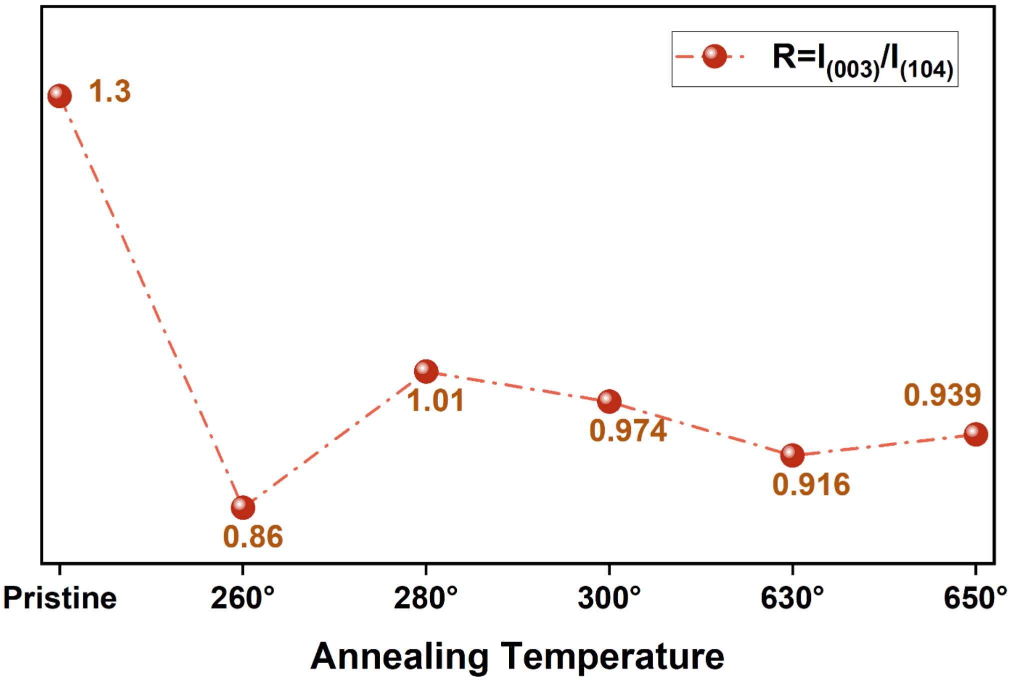

Figure S8: R-value from ex-situ XRD of P_NMC811, OV260_NMC811, OV280_NMC811, OV300_NMC811, OV630_NMC811 and OV650_NMC811 after 10 months in ambient atmosphere.

Table S2: Fitting parameters, binding energies (eV) and atomic (%) of XPS O1s Spectra of P_NMC 811, OV300_NMC811 and OV630_NMC811

P_NMC811

Name	Peak BE	FWHM eV	Area (P) CPS.eV	Atomic %
Lattice O	529.3	1.64	7842.72	5.83
ROLi	531	1.64	31278.71	23.27
Surface O/CO3/O-C=O	532	1.64	55706	41.48
C-O/O-C=O/OP(OR)3	533.4	1.64	18429.32	13.74

LixPFyOz	534.8	2.88	21000.16	15.67
OV300_NMC811				
Name	Peak BE	FWHM eV	Area (P) CPS.eV	Atomic %
Lattice O	529.4	1.64	2498.55	13.2
ROLi	531.24	1.64	8297.23	43.89
Surface O/CO3/O-C=O	532.29	1.64	6534.92	34.59
C-O/O-C=O/OP(OR)3	533.4	1.64	1174.84	6.22
LixPFyOz	534.8	2.88	395.83	2.1
OV630_NMC811				
Name	Peak BE	FWHM eV	Area (P) CPS.eV	Atomic %
Lattice O	528.88	1.63	14039.24	29.99
ROLi	531.15	1.64	20063.41	42.93
Surface O/CO3/O-C=O	532	1.64	10589.01	22.67
C-O/O-C=O/OP(OR)3	533.4	1.64	1523.06	3.26
LixPFyOz	534.8	2.88	531.94	1.14

Table S3: Fitting parameters, binding energies (eV) and atomic (%) of XPS C1s Spectra of P_NMC 811, OV300_NMC811 and OV630_NMC811

P_NMC811				
Name	Peak BE	FWHM eV	Area (P) CPS.eV	Atomic %
C-C/C-H	284.4	1.3	3037.92	33.7
C-O	285.54	1.39	1350.31	13.95
C=O/O-C-O	286.56	1.66	1094.11	16.18
O=C-O	288.1	1.48	837.16	7.88
CO3	289.55	1.58	2292.88	28.28
OV300_NMC811				
Name	Peak BE	FWHM eV	Area (P) CPS.eV	Atomic %
C-C/C-H	284.4	1.3	523.03	43.8
C-O	285.54	1.39	150.19	12.59
C=O/O-C-O	286.56	1.66	139.93	11.73
O=C-O	288.1	1.48	118.9	9.98
CO3	289.55	1.58	260.67	21.9
OV630_NMC811				
Name	Peak BE	FWHM eV	Area (P) CPS.eV	Atomic %
C-C/C-H	284.4	1.3	1623.99	28.34

C-O	285.54	1.39	614.29	10.73
C=O/O-C-O	286.56	1.66	389.62	6.81
O=C-O	288.1	1.48	282.6	4.94
CO3	289.55	1.58	2808.08	49.17

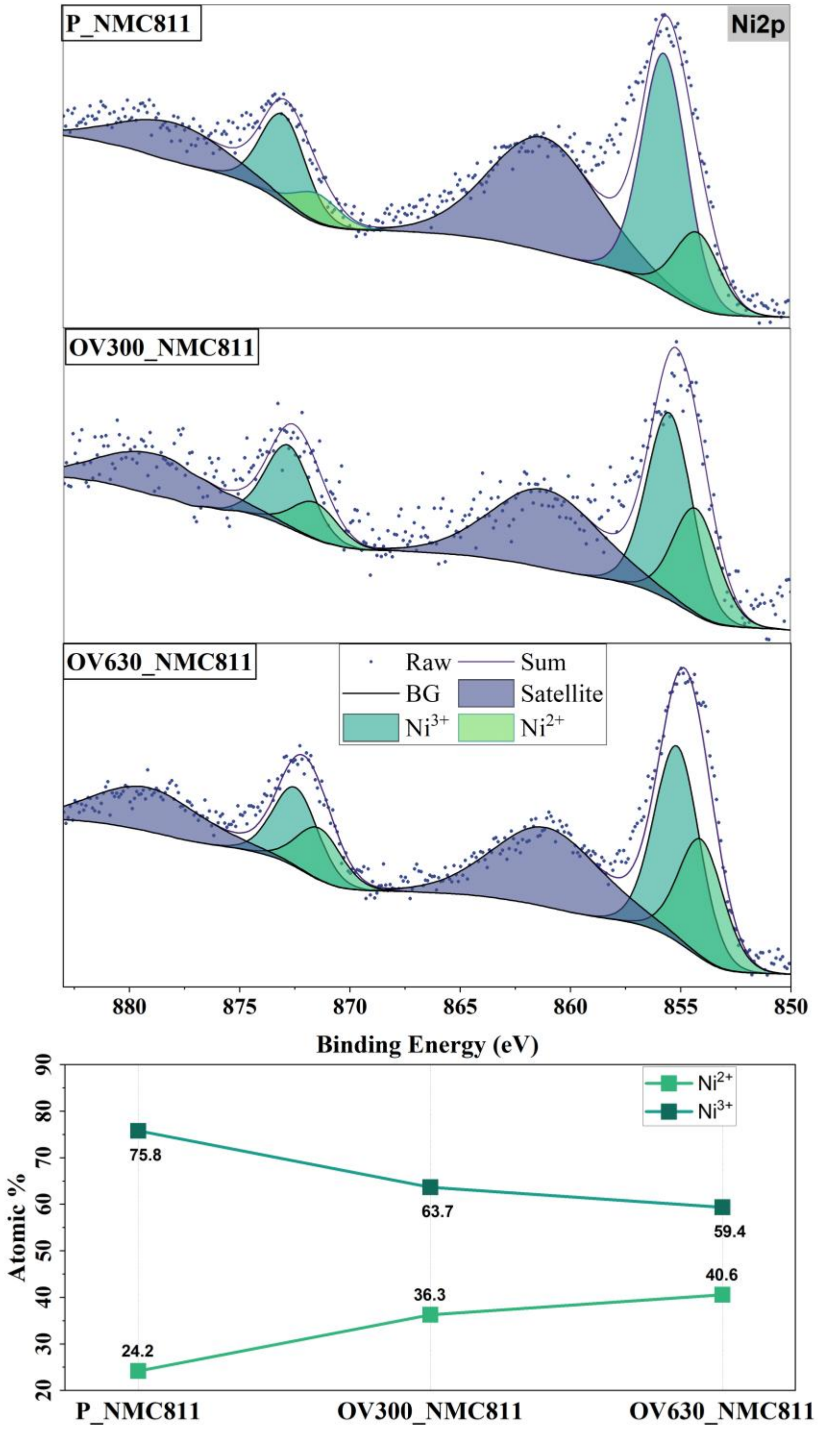

Figure S9: Ex-situ XPS spectra of Ni 2p for P_NMC811, OV300_NMC811, and OV630_NMC811. Deconvolution of peak reveals a progressive increase in the Ni^{2+}/Ni^{3+} ratio with higher annealing temperature, indicating enhanced surface reduction and formation of NiO-rich phases.

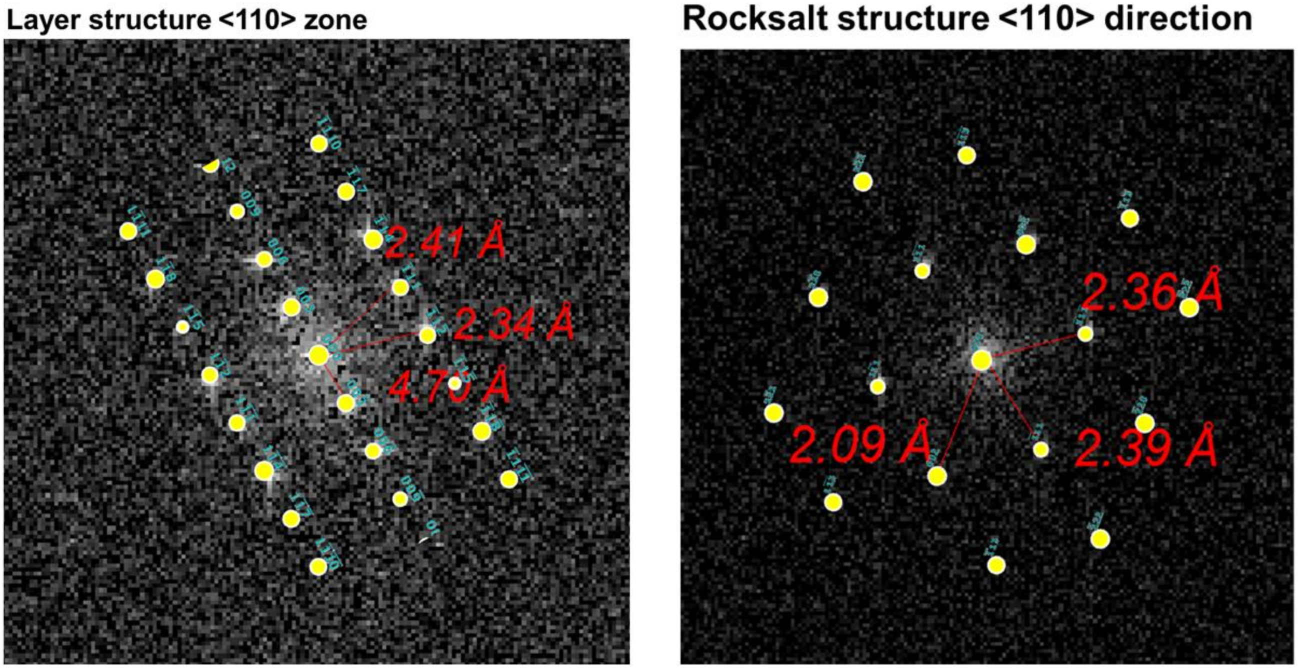

Figure S10. FFT of STEM images showing zone axes of different crystal structures in OV300_NMC811: (a) R-3m phase along the <110> direction, and (b) Fm-3m rocksalt phase along the <110> direction.

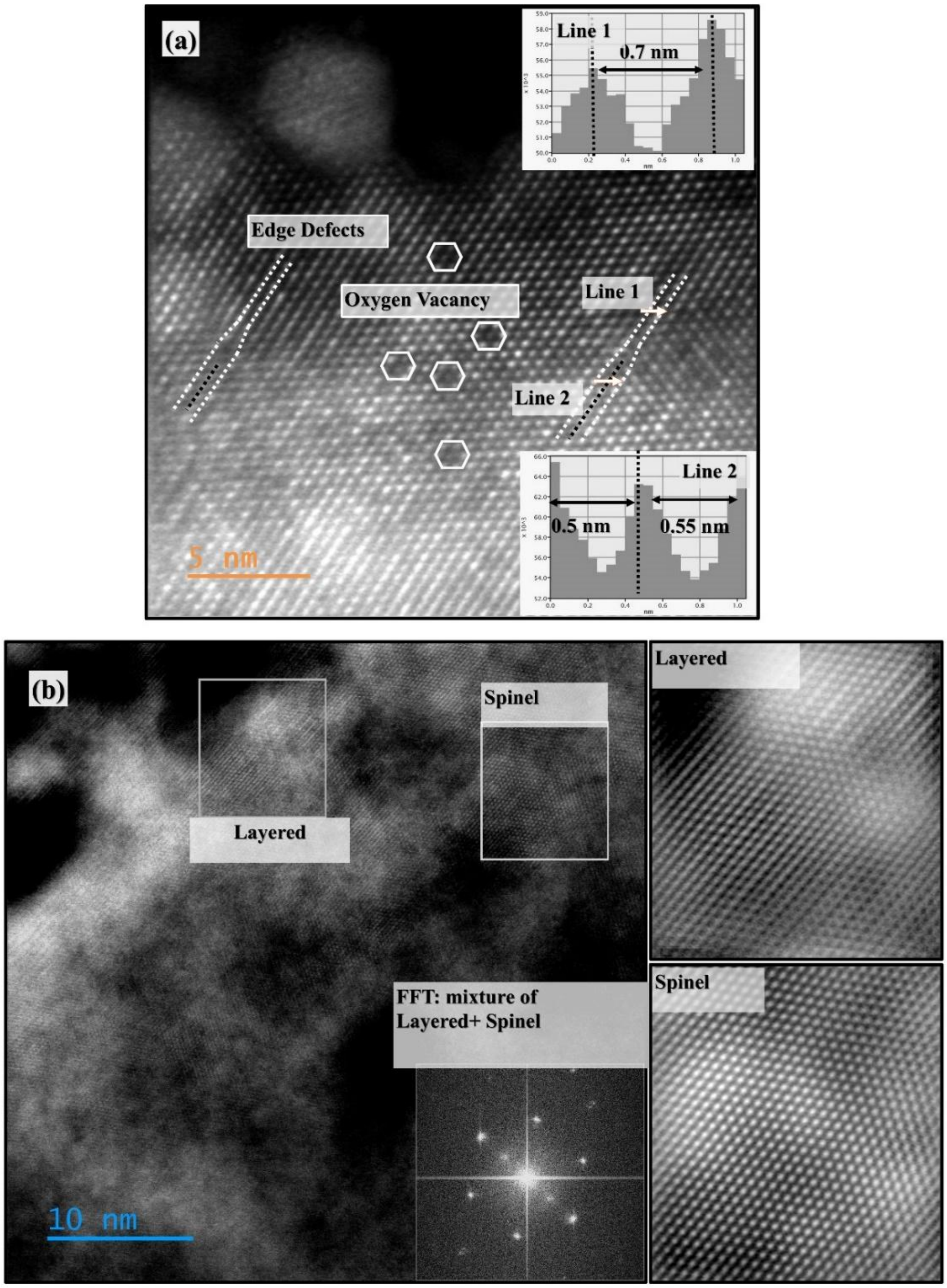

Figure S11: (a) STEM image of OV300_NMC811 shows a dimmed Ni atomic columns, potentially indicating Li migration into Ni sites during NiO or oxygen vacancy formation. The accompanying histogram highlights increased stacking faults.
(b) STEM image of P_NMC811 reveals a mixed-phase surface composed of layered and rocksalt domains, consistent with uncontrolled surface reconstruction.

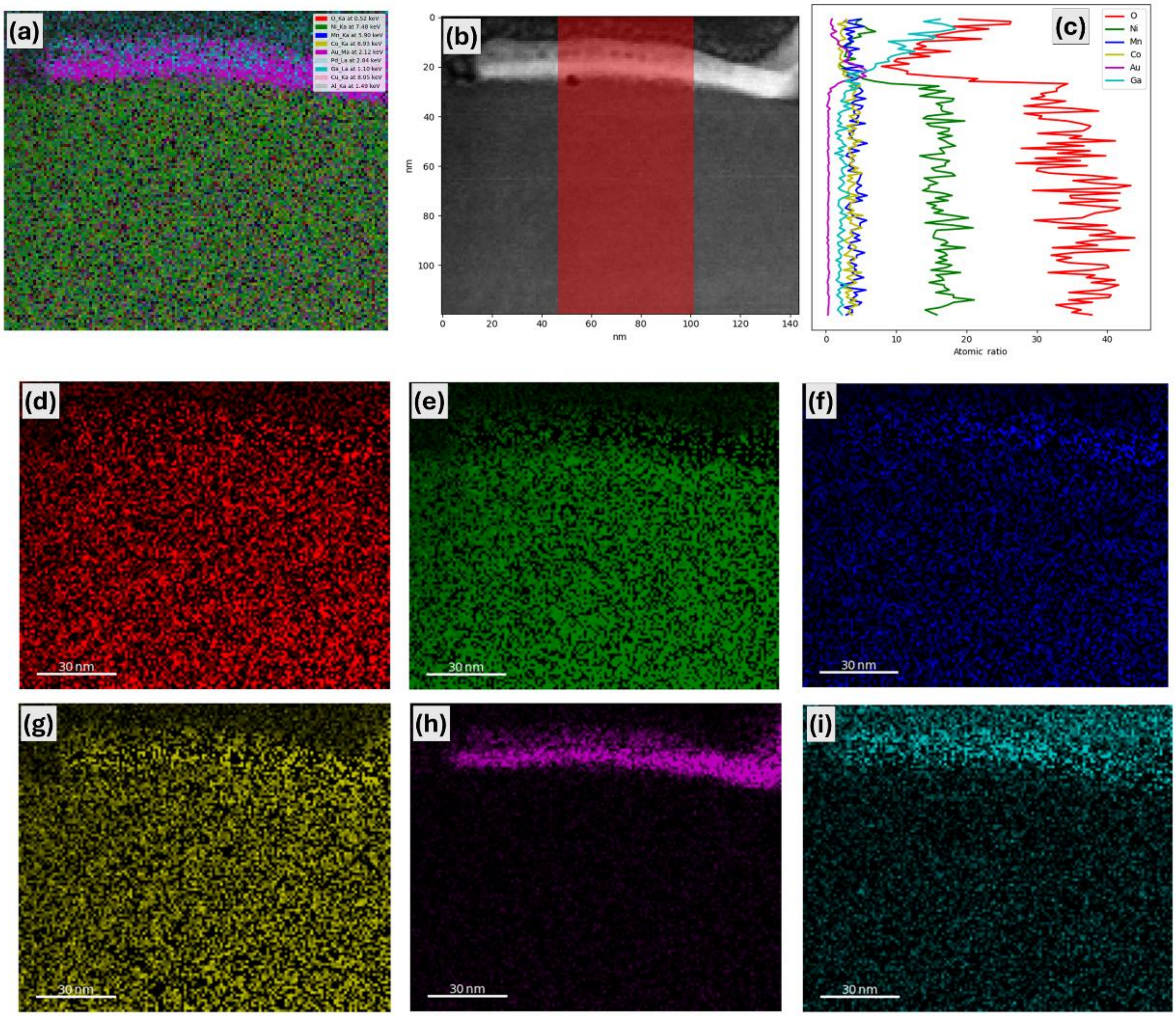

Figure S12. STEM-EDS analysis investigating surface damage potentially introduced during sample preparation: (a) overlapped elemental maps, (b) cross-sectional image, and (c) atomic ratios as a function of distance from the surface. Individual elemental maps of (d) O, (e) Ni, (f) Mn, (g) Co, (h) Au, and (i) Ga are shown. The absence of Ga and Au signals beneath the carbon and Au coating layers confirms minimal ion beam damage and effective sample preparation.

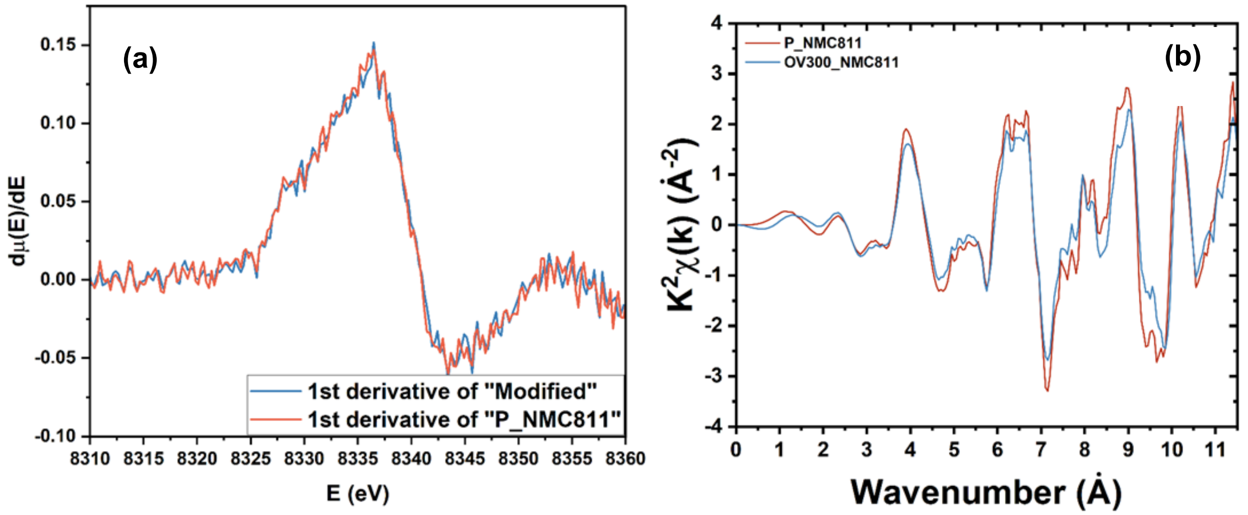

Figure S13. Hard X-ray absorption spectroscopy (h-XAS) data showing
(a) derivative plot of the Ni-K absorption edge, highlighting that there is no oxidation state change between modified and pristine sample, and
(b) corresponding k-space spectra used for extended X-ray absorption fine structure (EXAFS) analysis.

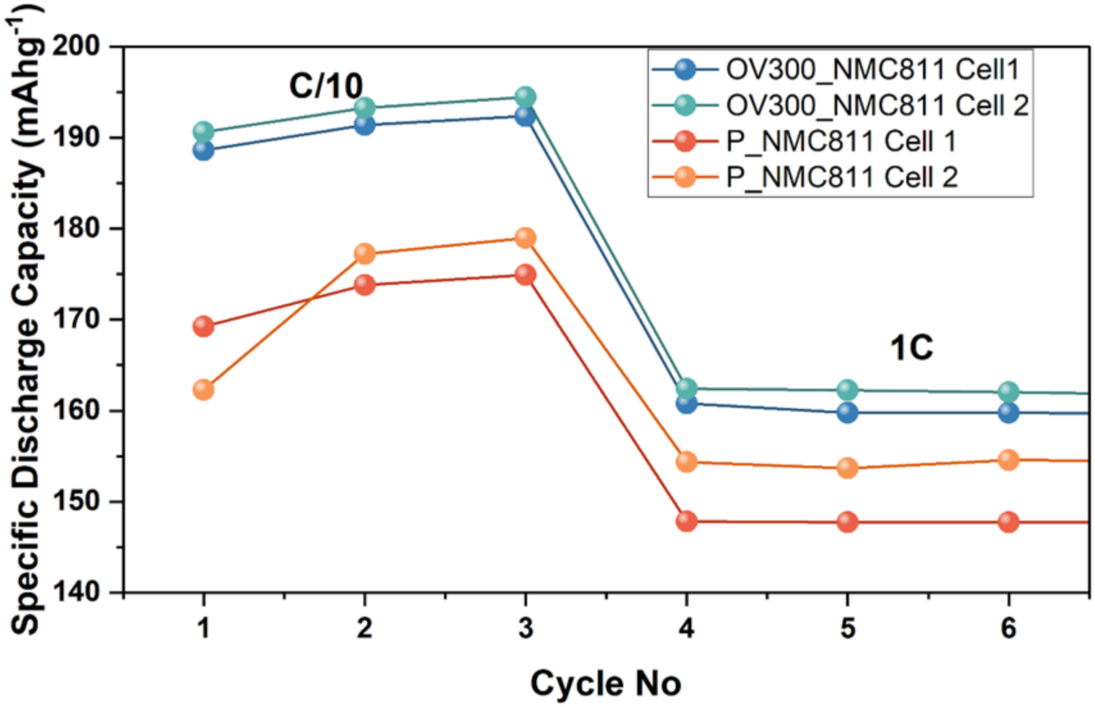

Figure S14. Two P_NMC811 and two OV300_NMC811 cells during three initial formation cycles at C/10

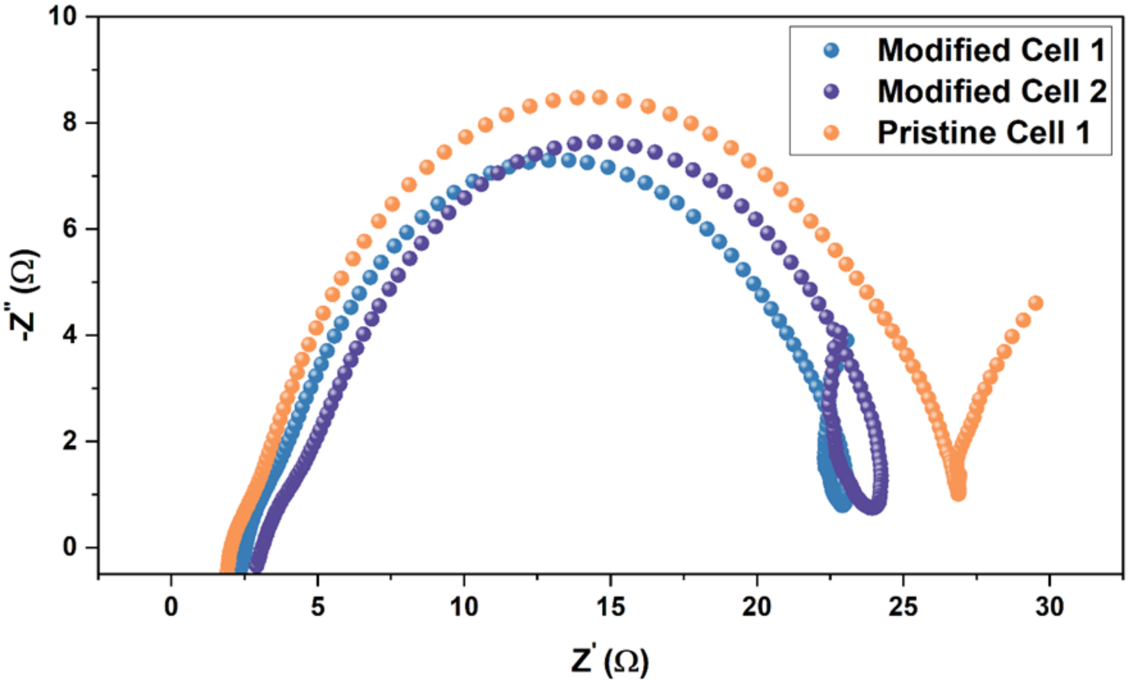

Figure S15. The negative capacitance loop at low frequency was tested for reproducibility using two independent cells OV300_NMC811 before cycling

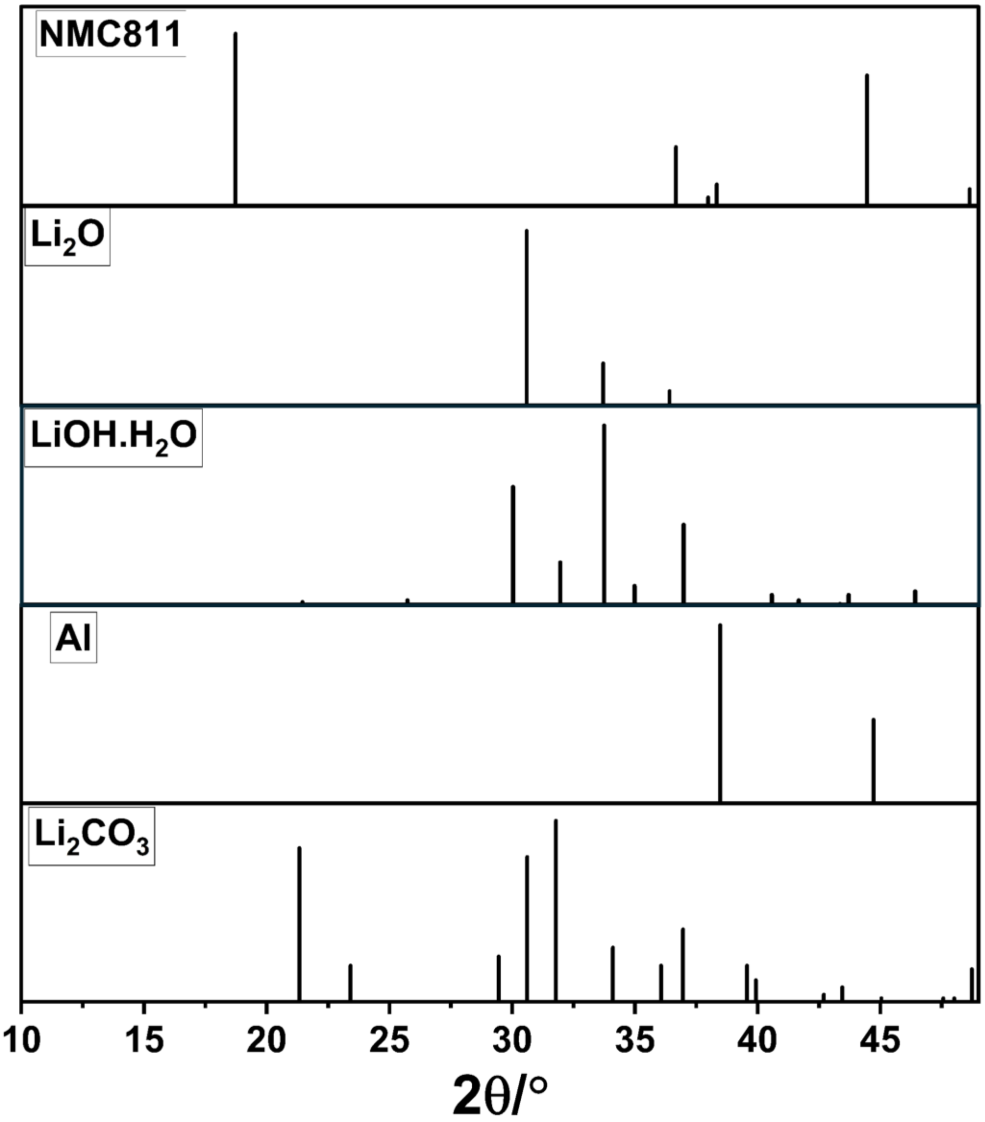

Figure S16. XRD patterns corresponding to NMC811, Li_2O, Al, Li_2CO_3, $LiOH.H_2O$-phases

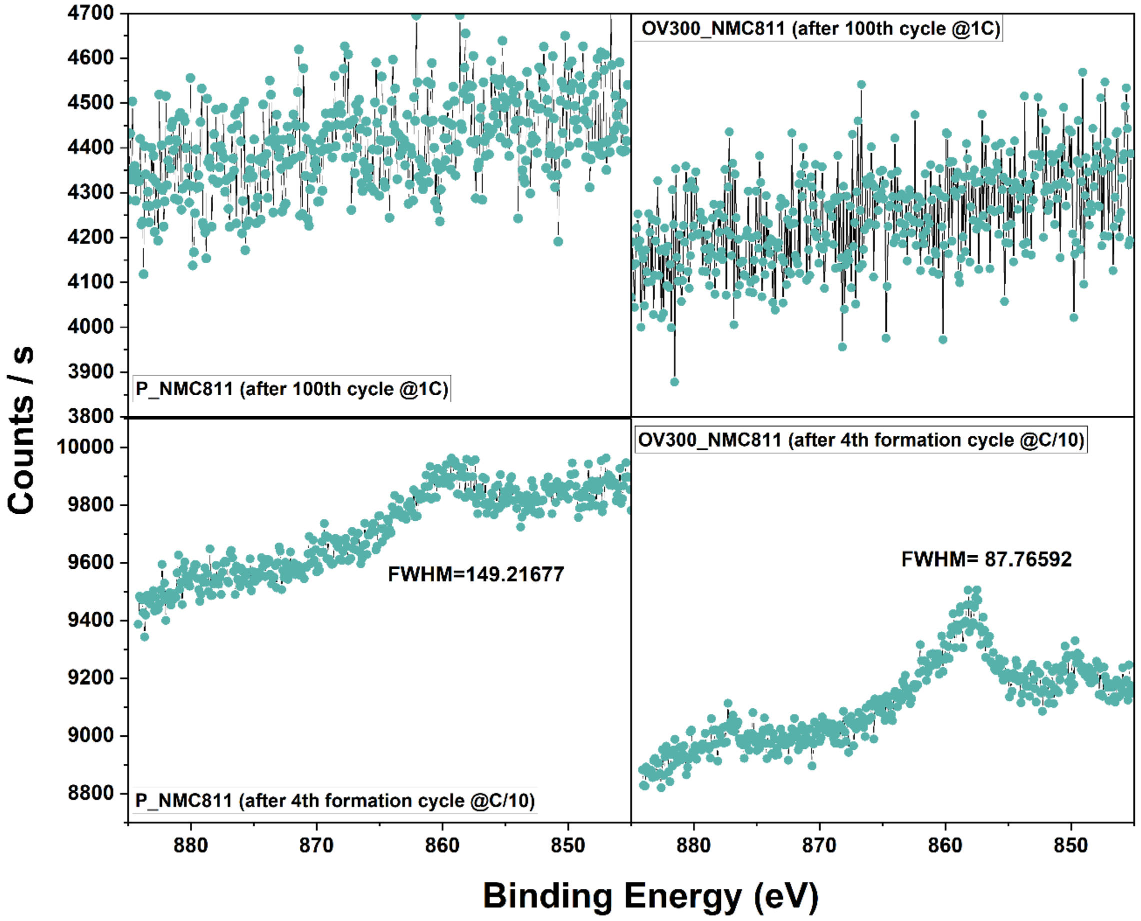

Figure S17: Post-cycling structural and surface characterization of P_NMC811 and OV300_NMC811: XPS Ni2p spectra after formation and 100th cycle respectively.

Refernceces:

[1] P. Giannozzi, S. Baroni, N. Bonini, M. Calandra, R. Car, C. Cavazzoni, D. Ceresoli, G. L. Chiarotti, M. Cococcioni, I. Dabo, A. Dal Corso, S. de Gironcoli, S. Fabris, G. Fratesi, R. Gebauer, U. Gerstmann, C. Gougoussis, A. Kokalj, M. Lazzeri, L. Martin-Samos, N. Marzari, F. Mauri, R. Mazzarello, S. Paolini, A. Pasquarello, L. Paulatto, C. Sbraccia, S. Scandolo, G. Sclauzero, A. P. Seitsonen, A. Smogunov, P. Umari, R. M. Wentzcovitch, “QUANTUM ESPRESSO: a modular and open-source software project for quantum simulations of materials” *Journal of Physics: Condensed Matter* **2009**, *21*, 395502.

[2] P. Giannozzi, O. Andreussi, T. Brumme, O. Bunau, M. Buongiorno Nardelli, M. Calandra, R. Car, C. Cavazzoni, D. Ceresoli, M. Cococcioni, N. Colonna, I. Carnimeo, A. Dal Corso, S. de Gironcoli, P. Delugas, R. A. DiStasio, A. Ferretti, A. Floris, G. Fratesi, G. Fugallo, R. Gebauer, U. Gerstmann, F. Giustino, T. Gorni, J. Jia, M. Kawamura, H.-Y. Ko, A. Kokalj, E. Küçükbenli, M. Lazzeri, M. Marsili, N. Marzari, F. Mauri, N. L. Nguyen, H.-V. Nguyen, A. Otero-de-la-Roza, L. Paulatto, S. Poncé, D. Rocca, R. Sabatini, B. Santra, M. Schlipf, A. P. Seitsonen, A. Smogunov, I. Timrov, T. Thonhauser, P. Umari, N. Vast, X. Wu, S. Baroni, “Advanced capabilities for materials modelling with Quantum ESPRESSO” *Journal of Physics: Condensed Matter* **2017**, *29*, 465901.

[3] S. Yousuf, M. M. Mridha, R. Magri, “Structures and electronic states of nickel-rich oxides for lithium ion batteries” *Mater Adv* **2024**, *5*, 2069–2087.

[4] H. Zhang, Y. Zhong, C. Ouyang, X. Gong, H. Xiang, "Theoretical study on the magnetic properties of cathode materials in the lithium-ion battery" *Journal of Chemical Physics* **2023**, *158*, DOI 10.1063/5.0137972.

[5] B. Ravel, M. Newville, "*ATHENA* , *ARTEMIS* , *HEPHAESTUS* : data analysis for X-ray absorption spectroscopy using *IFEFFIT*" *J Synchrotron Radiat* **2005**, *12*, 537–541.

[6] W. Kao-ian, R. Pornprasertsuk, P. Thamyongkit, T. Maiyalagan, S. Kheawhom, "Rechargeable Zinc-Ion Battery Based on Choline Chloride-Urea Deep Eutectic Solvent" *J Electrochem Soc* **2019**, *166*, A1063–A1069.
